# An Automated Approach to Assess Relative Galectin-Glycan Affinity Following Glycan Microarray Analysis

**DOI:** 10.3389/fmolb.2022.893185

**Published:** 2022-08-11

**Authors:** Alex D. Ho, Shang-Chuen Wu, Nourine A. Kamili, Anna V. Blenda, Richard D. Cummings, Sean R. Stowell, Connie M. Arthur

**Affiliations:** ^1^ Joint Program in Transfusion Medicine, Department of Pathology, Brigham and Women’s Hospital, Harvard Medical School, Boston, MA, United States; ^2^ Department of Biomedical Sciences, University of South Carolina School of Medicine Greenville, Greenville, SC, United States; ^3^ Department of Surgery, Beth Israel Deaconess Medical Center, Harvard Medical School, Boston, MA, United States

**Keywords:** galectin, microarray, dissociation constant, R studio, python

## Abstract

Numerous studies have highlighted the utility of glycan microarray analysis for the elucidation of protein-glycan interactions. However, most current glycan microarray studies analyze glycan binding protein (GBP)-glycan interactions at a single protein concentration. While this approach provides useful information related to a GBP’s overall binding capabilities, extrapolation of true glycan binding preferences using this method fails to account for printing variations or other factors that may confound relative binding. To overcome this limitation, we examined glycan array binding of three galectins over a range of concentrations to allow for a more complete assessment of binding preferences. This approach produced a richer data set than single concentration analysis and provided more accurate identification of true glycan binding preferences. However, while this approach can be highly informative, currently available data analysis approaches make it impractical to perform binding isotherms for each glycan present on currently available platforms following GBP evaluation. To overcome this limitation, we developed a method to directly optimize the efficiency of assessing association constants following multi-GBP concentration glycan array analysis. To this end, we developed programs that automatically analyze raw array data (kdMining) to generate output graphics (kaPlotting) following array analysis at multiple doses. These automatic programing methods reduced processing time from 32.8 h to 1.67 min. Taken together, these results demonstrate an effective approach to glycan array analysis that provides improved detail and efficiency when compared to previous methods.

## Introduction

Glycan binding proteins (GBPs) play crucial roles in various biological activities (Elola et al., 2007; Yang et al., 2008; Arthur et al., 2015a; Bochner and Zimmermann, 2015; Schnaar, 2015). These diverse functions are commonly dictated by specific interactions with glycan ligands (Rabinovich and Toscano, 2009). As a result, clear understanding of glycan binding specificity is key to elucidation of biological function ((Raman et al., 2005; Sato et al., 2009; Vasta, 2012). Given the often complex nature of glycan ligand synthesis, early studies often utilized relatively simple sugar substrates to probe GBP-glycan interactions (Cummings et al., 2009). However, as numerous studies suggest that subtle shifts of cell surface glycan modification are key to regulation of cell sensitivity to GBP activity, development of glycan microarrays representing extensive libraries of complex carbohydrate structures have provided key insight into GBP function (Blixt et al., 2004; Stowell et al., 2008a; McQuillan et al., 2019). In this way, glycan array analysis has enabled immense progress within the field of glycobiology by allowing assessment of GBP binding towards a number of complex glycan substrates simultaneously (Blixt et al., 2004; Stowell et al., 2008a; Song et al., 2009; Arthur et al., 2014; Arthur et al., 2015b; Gao et al., 2019). However, while glycan array analysis provides a wealth of information regarding GBP interactions with a wide variety of glycan substrates, glycan binding is often probed at only a single high concentration of GBP. This reflects challenging nature of microarray development and analysis, which remains time and resource demanding, often limiting analyses to a single concentration of a given GBP on an array. While this represents a reasonable method for the screening of glycan binding preferences for a given GBP, potential variations in the density of glycans printed on a glycan microarray may cause subtle inaccuracies in binding data. Often, this inaccuracy comes from the varied glycan density of different glycans on a printed array slide. While relatively minor, these differences, which may suggest varied printing efficiency across glycans, can result in less accurate glycan binding affinity comparisons. For this reason, we sought to establish whether binding analysis across multiple concentrations of GBP may enable relative binding affinity analyses that could serve to overcome inherent variability in the glycan printing process.

Assessment of GBP glycan array binding over a series of concentrations allows for estimation of maximal binding values (B_max_) and a relative dissociation constant (*K*_D_) (Perkins et al., 2012; Harding, 2021; Acipreste Hudson et al., 2022). Among GBPs analyzed for carbohydrate binding specificity, galectins represent some of the most studied. Galectins can be subdivided into three subgroups based on the quaternary structure of the protein (Verkerke et al., 2022). Each of the galectins analyzed in this study represent an example of each subgroup described including chimera type (hGal-3), prototype (hGal-7) and tandem repeat type (hGal-9) (Arthur et al., 2015a). Glycan binding was compared using a rank order approach at a single concentration compared to the relative affinity observed over a range of concentrations. We also included the C-terminal carbohydrate recognition domain (CRD) of hGal-3 (hGal-3C) and both the N and C-terminal CRDs of hGal-9 (hGal-9N and hGal-9C, respectively) for direct comparison to their full-length counterparts using this approach. To facilitate this work and improve the efficiency and productivity of GBP binding interrogation by glycan microarray analyses, while also reducing the possibility for human errors, we developed an automatic program, kdMining, to streamline the glycan-protein calculation and its saturation determination. This program was used to calculate B_max_, *K*_D_, *K*_A_., and % saturation (when saturation toward a given glycan did not occur) for each GBP analyzed accurately and efficiently. Furthermore, we developed a complementary program, kaPlotting, which processes some calculated outputs of kdMining, including relative *K*_A_ (for saturated binding) and %max (for unsaturated binding) into nested pie charts to enable more efficient glycan-protein interaction analyses and multi-GBP comparisons. Together these tools provide a useful method for analyzing GBP binding preference.

## Materials and Methods

### Sample Collection and Processing

Human galectin was expressed and stored as outlined previously (Wu et al., 2021a; Paul et al., 2022). Six previously published human galectin datasets, human Gal-3, Gal-3C, Gal-7, Gal-9, Gal-9N, and Gal-9C (Wu et al., 2021b PMID:35754739; Blenda et al., 2022), were used to evaluate the performance of kdMining and kaPlotting. Each purified galectin was directly labeled with Alexa Fluor™ 488 NHS Ester (Invitrogen) per manufacturer instructions. Labeled protein was tested for glycan binding on a Consortium for Functional Glycomics (CFG) glycan microarray slide as described previously at the indicated concentrations and scanned using GenePix 4000B microarray scanner (Stowell et al., 2007; Stowell et al., 2008; Stowell et al., 2009; Stowell et al., 2009). hGal-3 and hGal-3C were analyzed on CFG V3.0. Glycans common to both array versions were renumbered into a merged glycan list for analysis, while glycans present on only one of the two array versions were not considered in the current analysis. Imagene software (GenePix Pro 7) was used to generate integrated spot intensities that were then converted to a spreadsheet file (GPR, GPRS) containing the raw RFU data representing binding at each printed glycan location as outlined previously (Stowell et al., 2007; Stowell et al., 2008b; Stowell et al., 2009a; Stowell et al., 2009b; Wu et al., 2021c; Arthur et al., 2022; Stowell et al., 2022). RFU values for selected GBP-glycan pairs were then plotted manually using Graphpad Prism V9.0. Alternatively, GPR spreadsheets served as the input file for the kdMining program.

### Programming Method

kdMining was written using Python 3, a general-purpose and high-level programming language (Python Software Foundation, Wilmington, DE). Each input file had over 500 glycan samples along with their relative fluorescence units (RFU). The program first scanned all input spreadsheet files (GPR; GenePix Pro, San Jose, CA) in the designated directory once to ensure all input glycan sample files were present and in the correct format. The program can process and execute an infinite number of files (with multiple concentrations) given that the information does not exceed the capacity of the computer memory. All input files are stored within a dictionary, a data structure consists of key-value pairs in Python, while each key represents a single protein concentration that contains RFU values in the form of data frame. The program iterated through each protein concentration to calculate the average RFU and standard deviation (STDEV) from the 6 replicate spots for each sample. The program then combined all input files from the dictionary into a single data frame to determine percentage saturation (% max), average RFU values, dissociation constants (*K*_D_) and association constants (*K*_A_) for each glycan sample (Islam et al., 2012). The *K*_D_ was calculated by fitting a sample’s RFU values across all concentrations, while the *K*_A_ was calculated by taking the inverse of *K*_D_. For interactions that did not result in saturation, a % max value was provided by taking the absolute difference of a particular sample’s highest concentration RFU and the sample’s highest concentration RFU overall and calculating the percent difference. At the end of program execution, a spreadsheet file (Excel; Microsoft, Redmond, WA) of the samples’ calculated B_max_, *K*_D_, *K*_A_, and % max and their corresponding graphs were available.

kaPlotting was written using R, a programming language for statistical computing and graphics (R Foundation for Statistical Computing, Vienna, Austria). The program accepted the spreadsheet output from kdMining in the designated directory and processed one spreadsheet per execution. The program first parsed through the spreadsheet to ensure all needed columns, such as glycan chart number, glycan linkage and type, *K*_A_, and % max, are present in the file, then mapped all information onto the nested-circles. At the end of program execution, a total of three nested-circles were plotted based on the glycan structure: N-glycans, Lewis Antigen, and Sialic Acid are shown as examples. The program was designed such that each nested circle contains three layers of information: *K*_A_, glycan linkage, and glycan structure, from inner to outer, respectively. Since *K*_A_ and % max were numeric variables, they were presented with different color gradients from white to red and white to blue, respectively. The latest kdMining, kaPlotting, instructions, and a demo video can be found and downloaded from Mendeley Data (DOI: 10.17632/hrbtct6ryd.2).

## Results

**FIGURE 1:**
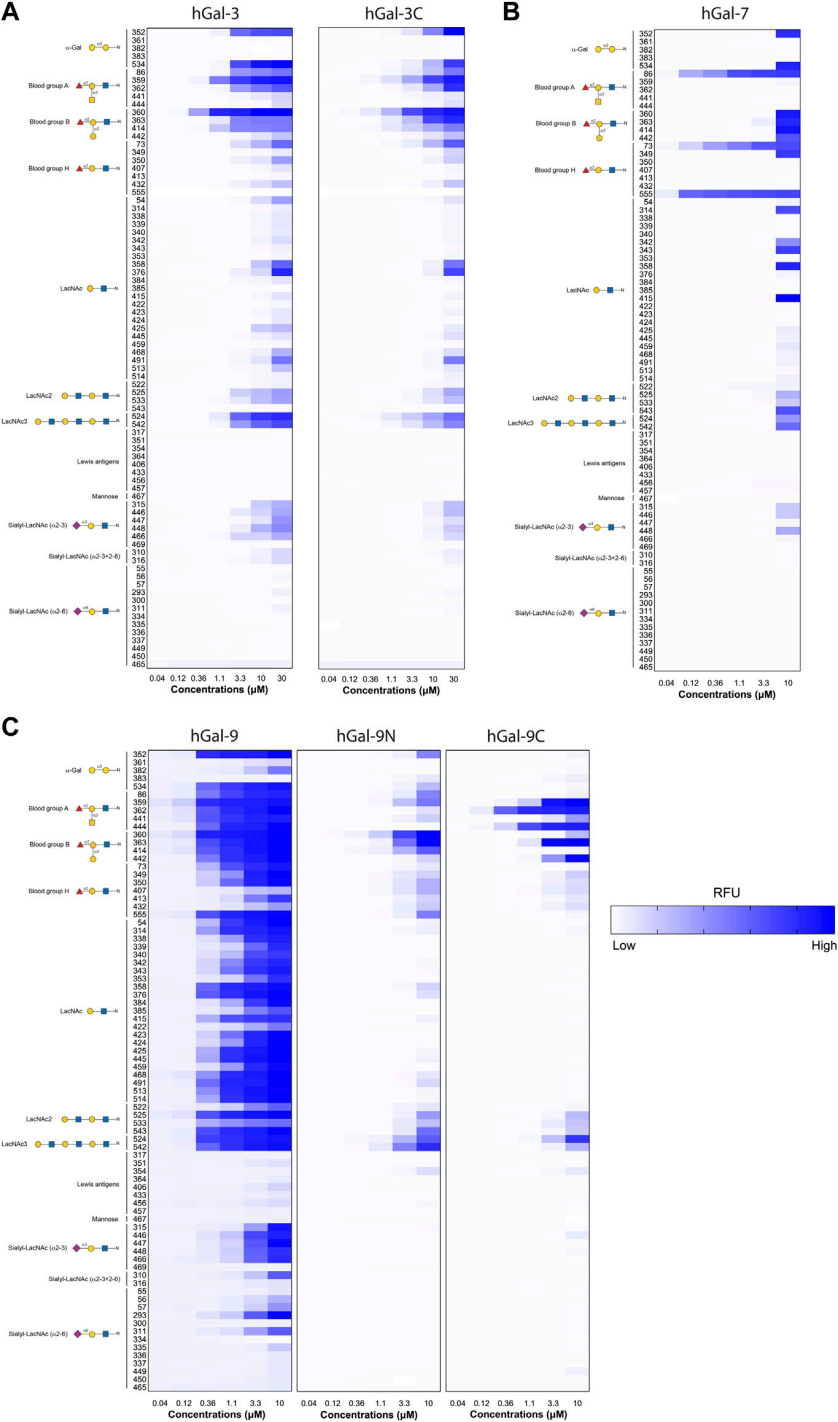
Examination of GBP-glycan binding affinity over multiple concentrations. **(A)** Heatmaps for the RFU values of Gal-3 and Gal-3C over a broad range of concentrations starting from 0.04 to 30 μM for antigens on N-Glycans. **(B)** Heatmap for the RFU values of Gal-7 over a range of concentrations starting from 0.04 to 10 μM for antigens on N-Glycans **(C)** Heatmaps for the RFU values of Gal-9, Gal-9N, and Gal-9C over a range of concentrations starting from 0.04 to 30 μM for antigens on N-Glycans. Detailed Symbol Nomenclature for Glycans (SNFG) structures and examples of glycans examined are shown on the left. The higher RFU value is shown with darker blue and lower RFU value shown in lighter blue.

To directly evaluate the relevance of multi-concentration screening of GBP-glycan interaction, we assessed glycan binding of human Gal-3, Gal-3C, Gal-7, Gal-9, Gal-9N, and Gal-9C over a range of concentrations (indicated in figure legends) as done previously (Stowell et al., 2008). We next visualized glycan binding as a heat map plot of RFUs detected in selected glycan binding. To better assess glycan binding patterns, complex glycan structures were grouped by common terminal glycan modification. When viewed this way, clear binding patterns emerge for each galectin tested, with lactosamine and blood group antigens appearing as the dominant terminal modification recognized (Figures 1A–C). These general binding capabilities can be appreciated by considering binding at the highest concentrations alone. However, evaluation of glycan binding at multiple concentrations reveals greater preference for particular terminal modifications as well as the important contribution of underlying glycan structural variation on true binding preference. In particular, hGal-7 binding provides a strong example of this limitation of assessment at only high concentrations as various glycan ligands appear equally preferred when considered at 10 µM alone, while dose dependent binding reveals exquisite specificity for particular presentations of the H antigen at lower concentrations (Figure 1B).

**FIGURE 2:**
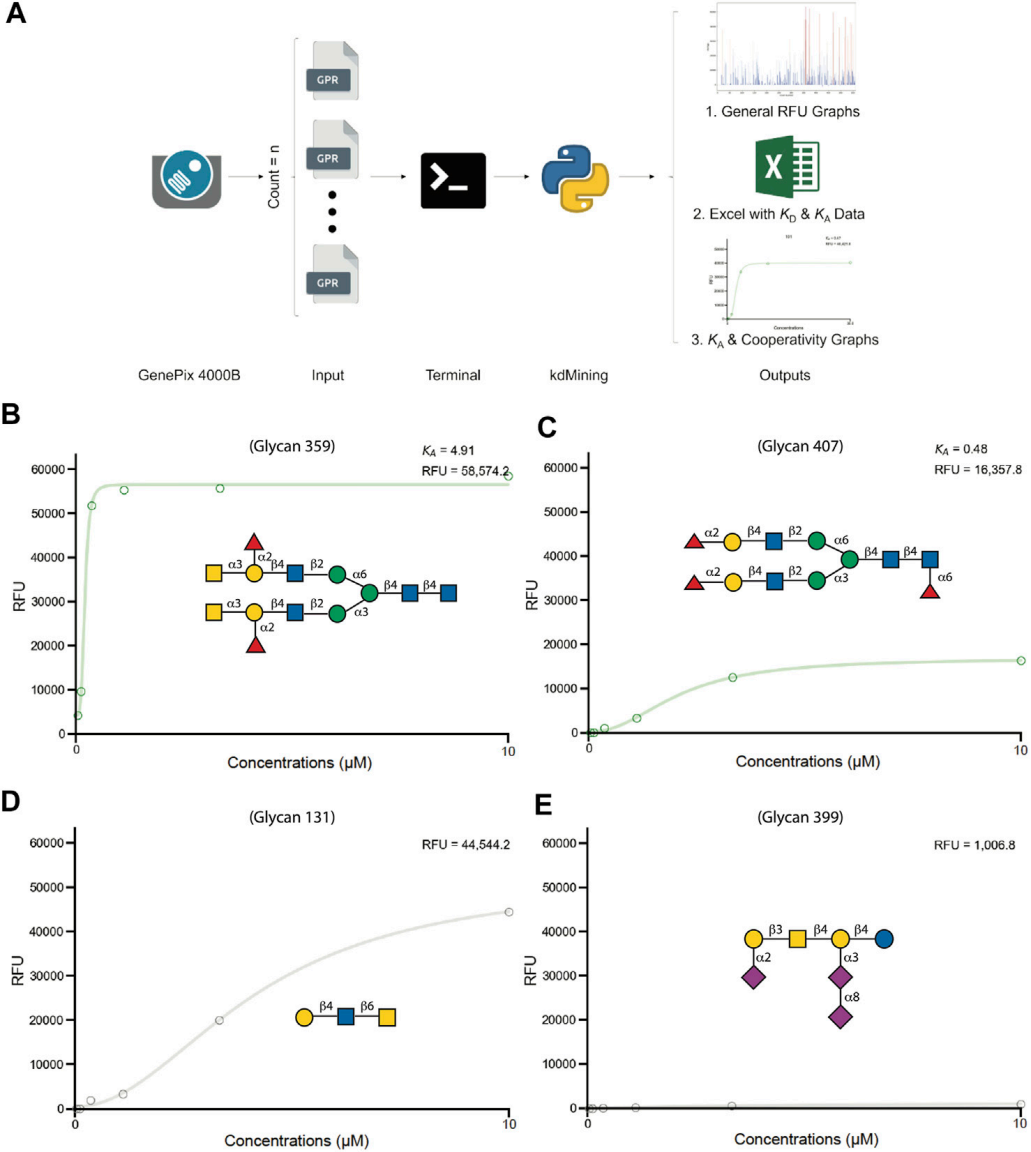
kdMining analysis for determining GBP-glycan binding affinity**. (A)** Overview of kdMining program. Once kdMining is executed in the machine’s terminal, all raw RFU gpr file inputs in the designated folder are processed automatically. Three types of output are generated: bar chart of RFU values for each protein concentration, Excel file of calculated *K*_D_ and *K*_A_, and fitted dose response curves for all glycan samples. **(B–E)** Demonstration of kdMining’s fitting graphs for hGal-9 binding at indicated concentrations (10uM, 3.3µM, 1.1µM, 0.36µM, 0.12µM, 0.04 µM). The fitted dose response curves for CFG glycan number 359 **(B)**, CFG glycan number 407 **(C)**, CFG glycan number 131 **(D)**, CFG glycan number 399 **(E)**. The calculated *K*_A_ and RFU value are shown on the upper right. The green and gray fitted lines represent saturated and unsaturated samples, respectively.

While assessment of glycan binding over multiple concentrations can offer significantly more insight than single concentration evaluation only, plotting data and evaluating binding patterns by manual calculation of average RFU for each dilution, followed by plotting the calculated RFU in a spreadsheet by density gradient (heatmap format) for visualization, represented large task. On average, to process and analyze one sample took 3.5 min, which translates to full dataset analyses of 555 glycans for all logistic patterns with curves requiring at least 1940 min (32.3 h). Additionally, even though this determination approach is commonly adopted, the possibility for human error and time demanded represented significant drawbacks to this method. To increase productivity and efficiency while reducing potential errors for *K*_A_ determination, we adopted a programming approach to expedite the process (Figure 2). To achieve this, we utilized an open-source programming language, Python 3, which offers the most substantial flexibility in time complexity and data structure. The output of the program includes general RFU graphs for each dilution, curve-fitting graphs for each sample, and calculated B_max_, *K*_D_, *K*_A,_ and % max binding for unsaturated glycan ligands in a spreadsheet file using the same approach as outlined previously (Blenda et al., 2022; Wu et al., 2021).

**FIGURE 3:**
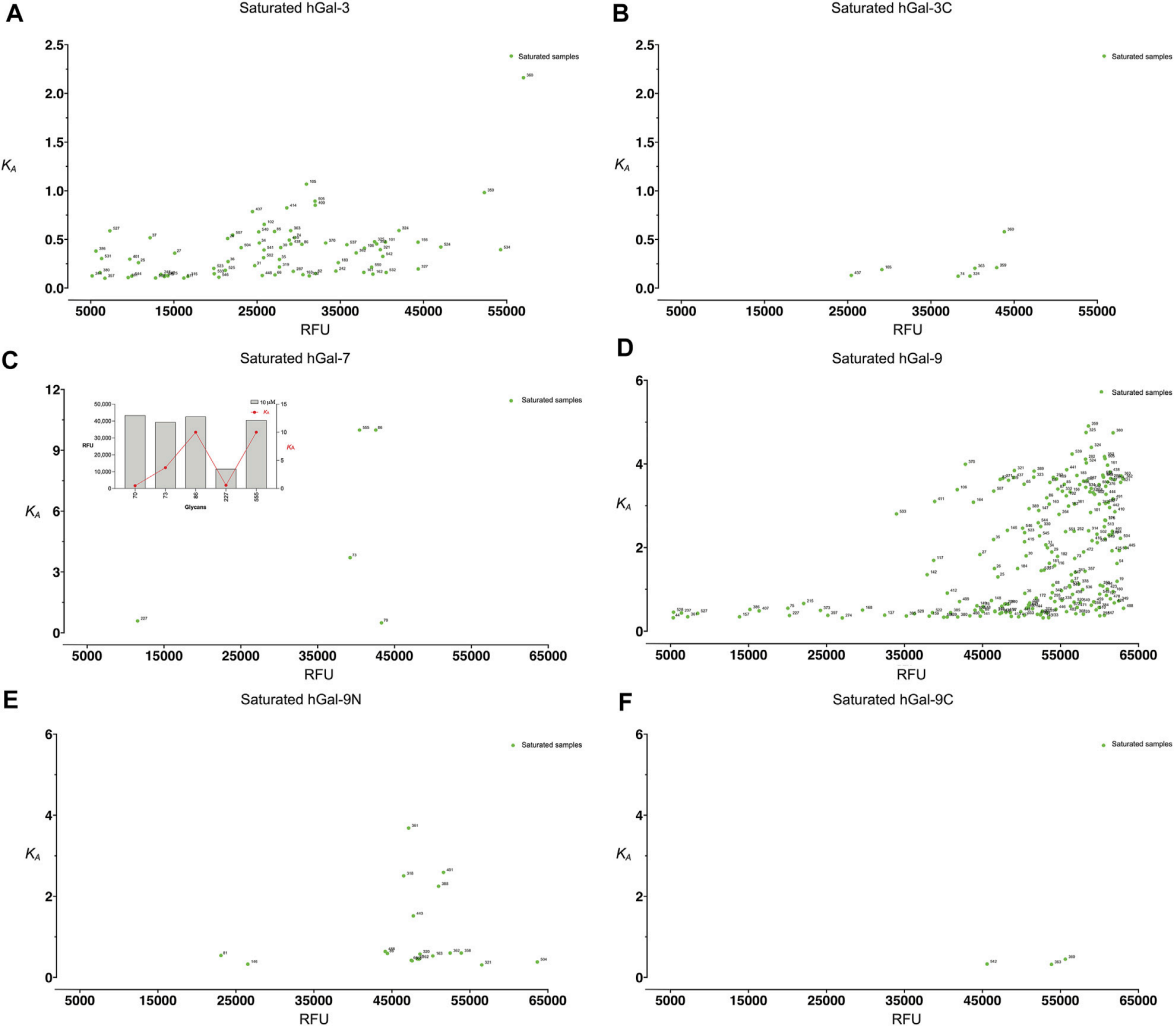
Examination of the distribution of saturated glycans for each galectin**. (A)** The saturated samples’ RFU values are plotted against *K*_A_ for Gal-3. **(B)** The saturated samples’ RFU values are plotted against *K*_A_ for Gal-3C **(C)** The saturated samples’ RFU values are plotted against *K*_A_ for Gal-7. A bar plot is presented with the five saturated samples along with their *K*_A_ and RFU values shown on the upper left. **(D)** The saturated samples’ RFU values are plotted against *K*_A_ for Gal-9 **(E)** The saturated samples’ RFU values are plotted against *K*_A_ for Gal-9N. **(F)** The saturated samples’ RFU values are plotted against *K*_A_ for Gal-9C.

**FIGURE 4:**
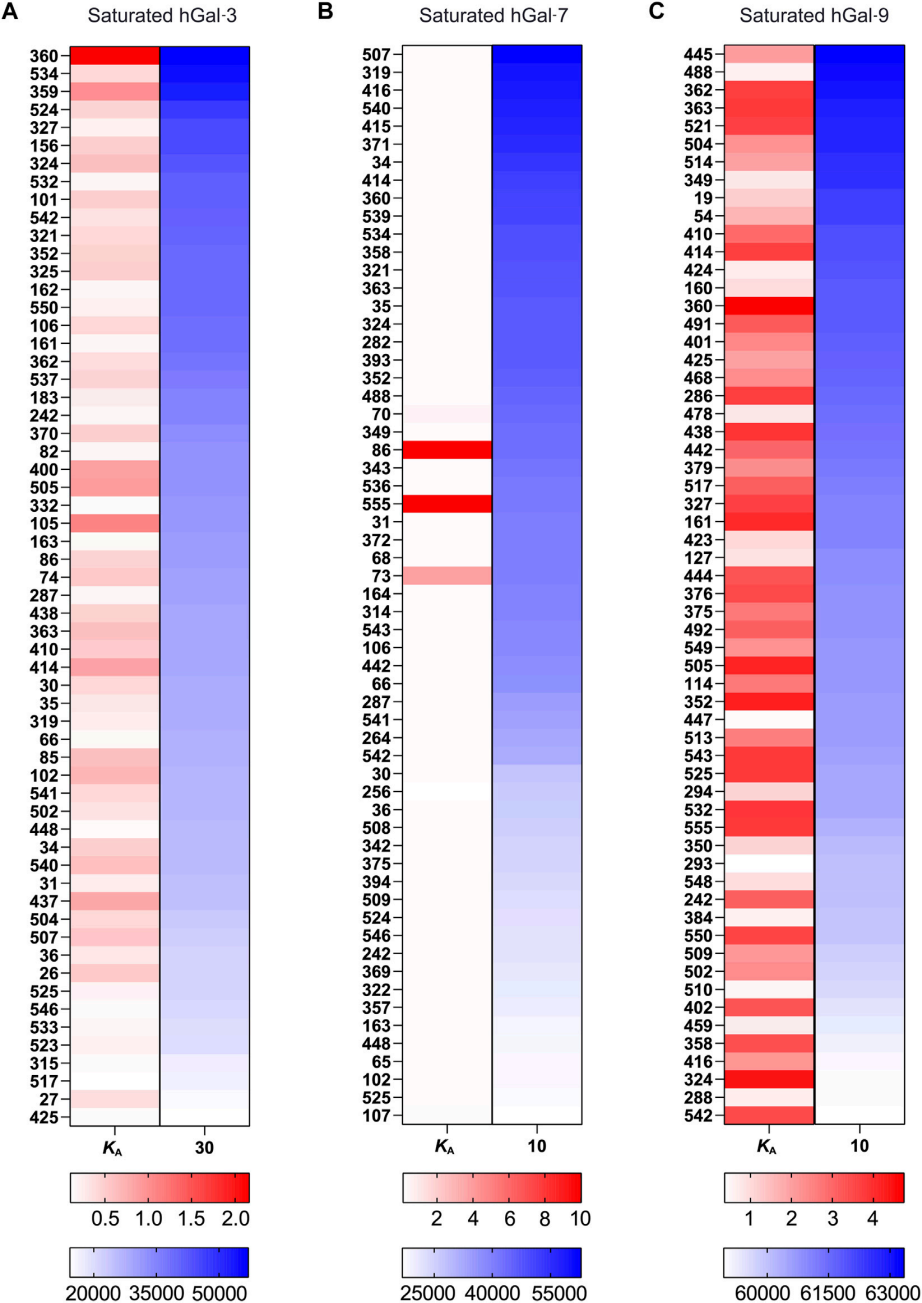
Examination of *K*_A_ and RFU correlation for selected galectin **(A)** A correlation map with the top 60 samples’ *K*_A_ and RFU values, sorted by RFU, for Gal-3. **(B)** A correlation map with the top 60 samples’ *K*_A_ and RFU values, sorted by RFU, for Gal-7. **(C)** A correlation map with the top 60 samples’ *K*_A_ and RFU values, sorted by RFU, for Gal-9. There are two sets of gradients: red and blue to indicate binding strength, for *K*_A_ and RFU, respectively. For both gradients, darker tint = higher calculated value, lighter tint = lower calculated value.

To demonstrate the programs’ performance, we fed the raw RFU data (GPR files) of human Gal-3, Gal-3C, Gal-7, Gal-9, Gal-9N, and Gal-9C into kdMining and got their corresponding calculated average B_max_, *K*_D_, *K*_A_, and % max (Supplementary Table S1) This program automatically produces individual RFU plots for each glycan represented on the array, with fitted curves and estimated *K*_A_, as well as the B_max_ for each plot. Each sample’s concentrations were plotted against their calculated average RFU values and fitted to a dose response curve. For demonstration purposes, we plotted average RFU values of Gal-9 and presented the fitted curves for several Gal-9 bound glycans. A green fitted line indicates that GBP binding over the multiple tested concentrations resulted in glycan binding saturation, while a gray fitted line indicates GBP binding that occurred over the multiple tested concentrations tested, but for which saturation was not achieved. To further investigate the relationship between RFU values obtained at the highest concentration tested as a rank order approach and *K*_A_ values obtained for the saturated samples, we plotted the RFU at the highest concentration tested against *K*_A_ for all galectins analyzed. Among all galectins, Gal-3 and Gal-9 binding resulted in saturation of many more glycans when compared to Gal-3C, Gal-7, Gal-9N, and Gal-9C (Figure 3). Finally, to reassess the effectiveness of kdMining in identifying accurate preferential GBP binding, we next plotted *K*_A_ values against maximum RFU for the top 60 bound glycans. Glycans were listed in order from highest to lowest maximum RFU using the rank order approach and corresponding *K*_A_ values were also shown as calculated by kdMining. Parallel analysis of *K*_A_ and maximum RFU revealed a poor match overall between *K*_A_ and maximum RFU (Figure 4). This analysis was also completed with glycans ranked by *K*_A_, showing similar results (Supplementary Figure S1). Importantly, preferential binding indicated by *K*_A_ calculations within our program matched the highest binders identified by manual analysis of binding at multiple concentrations (Figure 1).

**FIGURE 5:**
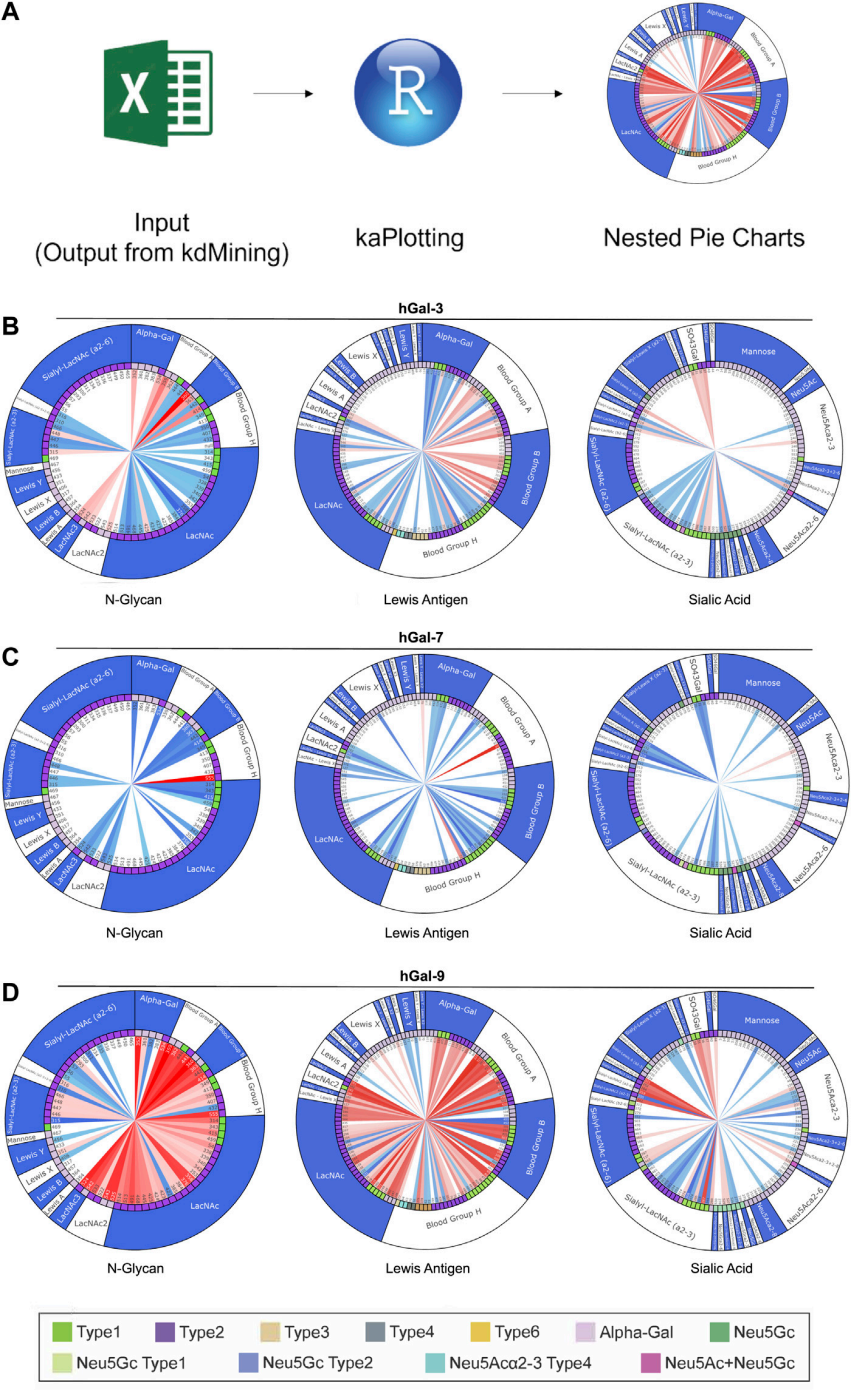
Demonstration of kaPlotting’s nested pie charts**. (A)** Overview of kaPlotting intakes and processing steps. A total of three three-layer-nested pie charts that consist of *K*_A_, glycan linkage, and glycan structure are generated. **(B–D)** Nested pie charts shown for Gal-3 **(B)**, Gal-7 **(C)** and Gal-9 **(D)**. Charts are grouped by N-Glycan, Blood Group Antigen, and Sialic Acid, and plotted using kaPlotting. Each nested pie chart has three layers of information: *K*_A_, glycan linkage, and glycan structure, from inner to outer, respectively. There are two sets of gradients: red and blue, that represent *K*_A_ and % max respectively on the inner circle. For both gradients, darker tint = higher calculated value, lighter tint = lower calculated value. Legend at the bottom shows the glycan linkage (second layer on the nested pie charts). Type 1: Type 1 LacNAc and type 2: Type 2 LacNAc.

Although kdMining was able to determine useful information regarding glycan-protein interactions, having the calculated values alone made it difficult to visualize the differences in the level of saturation and binding affinity when comparin multiple galectins. To address this limitation, we used an open-source programming language, R, which is known for its aesthetic graphing and plotting and strong statistical analysis to create a second programming step, kaPlotting, for data presentation (Figure 5). This program directly intakes the spreadsheet output from kdMining, and parses through the calculated values once to ensure all information is present. After each program execution, a total of three three-layered nested pie charts were drawn. Each nested pie chart consisted of similar families of glycan structures, and the layers showed the calculated *K*_A_, glycan linkage, and glycan structure for all glycan structures, from inner to outer, respectively. For demonstration purposes, we selected three pie charts: each of which primarily focus on N-glycans, blood group antigens and related structures, and sialic acid containing glycans, that had the most bindings for human Gal-3, Gal-7, Gal-9, Gal-3C, Gal-9N, and Gal-9C (Figures 5B–D, Supplementary Figure S2A–C). Within the *K*_A_ layer, there are two sets of gradients: red and blue. Red gradients indicate the *K*_A_ value for saturated samples, while blue gradients represent % max for unsaturated samples, and white indicates that no significant binding was detected. Since the glycan linkage and glycan structure layers contained categorical variables, discrete color schemes were used. As shown in the nested pie charts, Gal-3 bound to more glycan samples compared to Gal-3C, and its proportion of having saturated binding was also higher than human Gal-3C (Figure 5B, Supplementary Figure S2A). Gal-7 had much more unsaturated samples than the saturated ones (Figure 5C). Gal-9 bound to and saturated with a great number of glycans compared to Gal-9N and Gal-9C (Figure 5D, Supplementary Figure S2B, C).

**FIGURE 6:**
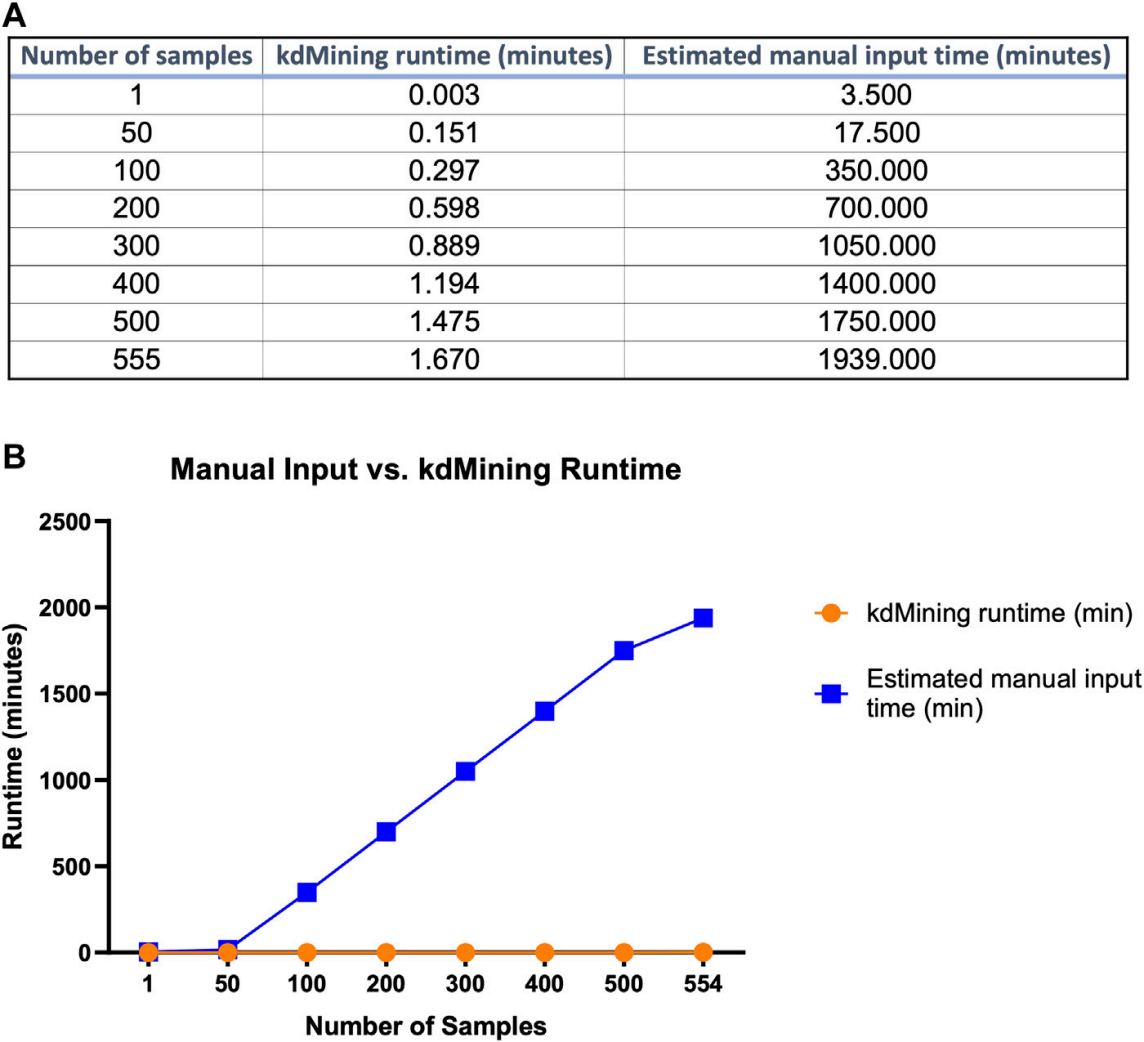
Execution time comparison of kdMining and manual approach**. (A)** To process and analyze 555 samples, kdMining takes less than 1.67 min, whereas the manual approach takes more than 32.3 h. **(B)** Both kdMining and the manual approach have a linear runtime, but kdMining can process information at a much faster rate.

While the information provided by kdMining and kaPlotting yielded accurate and efficient evaluation of GBP-glycan binding, we next wanted to evaluate the reduction in turnaround time needed to obtain *K*_A_ results from raw RFU values using KdMining. To accomplish this, we created different sizes of datasets and conducted multiple trials to test the analysis time between these two approaches (Figure 6). A runtime table was included to demonstrate the time required for each approach to execute the given number of samples (Figure 6A). As shown in the graph, even though both approaches had a linear runtime, the manual approach would take roughly 600 times longer on average than our approach using kdMining (Figure 6B). To analyze one sample, the runtimes of the manual approach and kdMining are 3.50 and 0.01 min, respectively. Furthermore, when the number of samples increased to 555, the manual approach took more than 1939 min (32.3 h), while kdMining took less than 1.7 min to complete the same amount of work. Based on these results, kdMining appears to be an effective and efficient approach to analyzing *K*_A_ and binding affinity following glycan microarray analysis.

## Discussion

Given the post-translational nature of glycan modifications (Cummings, 2009), defining GBP interactions with distinct glycan determinants has been challenging (Johannes et al., 2018). A variety of approaches, including isothermal calorimetry, surface plasmon resonance, equilibrium dialysis, fluorescence polarization and frontal affinity chromatography, have been used to measure the actual affinity of a given GBP with a specific glycan ligand (Ahmad et al., 2004; Brewer, 2002; Morris et al., 2004; Sörme et al., 2004; Hirabayashi et al., 2002; Leppänen et al., 2005; Mehta-D’souza, 2015). While each of these methods certainly has advantages over the approach outlined in the present work, there are significant challenges to using these strategies in a high throughput manner. Glycan array technology appeared to solve some of the throughput limitations of prior approaches by increasing the number of glycan determinants that can be analyzed following incubation with a single GBP(Rillahan and Paulson, 2011; Gao et al., 2019). Glycan microarrays also allow analyses to be achieved with very small amounts of glycan material, expanding the use of source materials for more GBP analyses (Rillahan and Paulson, 2011).

Despite the success of microarray analysis, common strategies employed in array analysis certainly have limitations. Most glycan array analysis is performed at a single GBP concentration, followed by rank order analysis to determine the glycans that appear to possess the highest affinity interaction with the GBP examined (Jonas, 1975; Delso et al., 2016). This approach has simplified glycan binding analysis and in so doing, provided a wealth of information regarding the key glycan determinants bound by individual GBPs(Bochner et al., 2005; Stevens et al., 2006; Carlsson et al., 2007; Stowell et al., 2010; Stowell et al., 2014; Wesener et al., 2015; Noll et al., 2016). However, possible glycan printing irregularities between slide manufacturing attempts or even intrinsic differences in printing efficiencies between individual glycans themselves can result in variation in the amount of glycan printed. This raises the possibility that differences in glycan binding achieved following GBP incubation could in part be influenced by the glycan print density and not the relative affinity of the GBP for the individual glycan. This can result in variation in the apparent strength of binding that is not a consequence of intrinsic glycan preference. As batch-to-batch glycan array printing can differ with respect to glycan printing efficiency, it can be difficult to normalize results to reduce bias introduced by uneven glycan printing; no positive control for glycan presentation and printing efficiency exists for every glycan. As a result, the use of single GBP concentration approaches, while capable of providing important insight into general binding capabilities for a given GBP, may miss more subtle GBP binding preferences. Such binding preferences may be important biologically, as relatively simple glycan modifications may alter GBP-glycan binding dynamics in ways that have real consequences on the overall outcome of GBP binding to a cell surface (Carlsson et al., 2007; Stowell et al., 2008c).

In an effort to overcome inherent limitations both in glycan microarray manufacturing and single concentration approaches to GBP glycan binding analysis, we and others have examined GBP binding over a range of concentrations (Stowell et al., 2008a). Using this approach, the B_max_ not only provides a more accurate reflection of the relative amount of a particular glycan that may be present, but also can be used to extrapolate a binding isotherm and *K*_D_ value to provide a relative affinity constant for GBP interactions with a given glycan (Garcia et al., 2014; Alhazmi, 2018; Huang et al., 2021). In doing so, the relative affinity of a GBP for a given glycan can be determined regardless of unintended variations in the amount of glycan printed. In this way, relative *K*_D_ assessment allows analysis approaches to more effectively control for glycan print variation while also providing a more accurate measure of the relative binding affinity of a GBP for a glycan ligand. The utility of this approach can be appreciated by the relative lack of correlation between *K*_A_ values and the maximum RFU obtained for each galectin analyzed. Thus, using this approach, more accurate assessments of binding affinity can be obtained. Importantly, using this approach, predictions regarding glycan binding specificity have accurately predicted actual glycan binding to cell surface glycans, providing important insight into previously unrecognized activities of a given galectin (Stowell et al., 2008a; Blenda et al., 2022).

Despite the possible utility of relative affinity measurements following glycan microarray analysis, the overall process required to analyze data in this manner is resource intense. Current programing packages for array analysis are designed to integrate spot intensity into an RFU value, which can be exported into an excel file for easy initial evaluation of glycan binding. This approach has been used for over a decade to assess glycan binding and can provide a rank order analysis of glycans bound by the GBP analyzed. However, for B_max_ and *K*_A_ value extrapolation of array data obtained over a range of GBP concentrations, there is no readily available automated approach capable of directly interfacing with raw microarray output data to generate these values. As a result, B_max_ and *K*_A_ value determinations are limited to either manual calculations based on array analyses, which often requires custom configuration of an excel platform, or the employment of commercially available software such as Prism or Sigmaplot. These later programs are user-friendly and can readily assist in B_max_ and *K*_A_ calculations. However, the manual input of data and limitations surrounding the number of glycans analyzed within an analysis file can make this approach cumbersome and vulnerable to common errors introduced by human data entry, especially when considering possible GBP interactions with over 500 individual glycans are analyzed. To facilitate this process, we generated kdMining, which allows *K*_D_ values to be directly extrapolated from raw microarray output data within a fraction of the time required for manual input. Using this approach, the relatively binding affinity toward individual glycans can be readily assessed within a fraction of the time required for manual data entry.

In addition to challenges assessing the actual binding affinity toward a given glycan, the sheer volume of data obtained following glycan microarray analysis can make data analysis difficult to manage and interpret. Rank order analysis does provide the highest binding glycans, but it fails to allow equally accessible analysis of those glycans to which a given GBP fails to bind. Negative binding outcomes are often just as critical as positive interactions as these data can provide critical information regarding the impact of key glycan binding determinants required for glycan binding (Carlsson et al., 2007). To facilitate *K*_A_ interpretation and therefore overall GBP binding specificity, we developed kaPlotting, which bins glycan binding data accordingly to common glycan determinants in order to facilitate interpretation of possible differences in GBP binding due to subtle variation in glycan presentation. By configuring the data in this manner, distinct GBPs can be directly compared and visually assessed. However, when glycan binding saturation does not occur, but interactions are present, these binding events are acknowledged through a distinct color code using the percent maximal binding similar to what was previously used to perform rank order analysis of glycan binding. By separating *K*_A_ and %max binding in this manner, higher affinity interactions can be observed in red, while variation in lower affinity binding can still be perceived within shades of blue. By combining this approach with recently developed analytical tools for glycan array analyses, overlapping and distinct glycan binding preferences between unique GBPs begin to become apparent (Mehta and Cummings, 2019).

In summary, the glycan binding profiles established following glycan binding analysis over a range of concentrations on existing glycan microarray technology has been shown to be a useful strategy in assessing GBP binding preference. However, limitations in the time required for analysis have prevented this approach from being commonly employed. Use of the kdMining and kaPlotting tools may aid in this analysis, allowing relative affinities to be obtained following GBP analysis towards hundreds of glycan determinants. Additionally, the developed programs could likewise be applied to a variety of microarray formats such as protein microarrays and DNA microarrays. These microarrays can be manufactured with the same microarray printer as the glycan microarray and analysis is often achieved using a similar approach. It should be noted that despite the possible advantages of coupling *K*_A_ calculations with glycan microarray analysis, this approach simply provides a *relative K*_A_ value. Actual *K*_A_ values will require confirmatory studies using more refined approaches, as outlined previously (Brewer, 2002; Hirabayashi et al., 2002; Ahmad et al., 2004; Morris et al., 2004; Sörme et al., 2004; Leppänen et al., 2005), to detail the binding affinity of a GBP toward individual glycans. Furthermore, there are clearly caveats to this approach and the impact of antigen printing densities and additional consideration could certainly influence the overall apparent binding affinity observed for a given GBP as stated previously (Zhang et al., 2010; Godula and Bertozzi, 2012; Tsouka et al., 2021). While the purpose of this study was to provide an automated approach to analyzing relative GBP binding affinities towards immobilized glycans in an array format, this is still a screening approach and exhaustive analysis of GBP glycan binding preferences often requires the utilization of multiple modalities, including surface plasmon resonance, isothermal calorimetry, fluorescent polarization, and other approaches (Brewer, 2002; Stowell et al., 2008a; Kumar et al., 2019), to appreciate the full binding characteristics. As these approaches often require higher concentrations of glycans or glycan modifications, binding isotherms generated following array analysis can provide an important triaging approach to determine which glycan determinants warrant additional consideration using more refined approaches. Equally important, array data and more refined approaches designed to define the glycan binding specificity of a given GBP require critical confirmatory experiments to determine whether binding affinities and overall specificity observed translate to glycan binding on a cell surface. By using combined approaches of array analysis, additional biochemical studies and ultimately biological approaches, a picture of the binding specificity of a GBP can emerge that provides important insight into its overall function.

## Data Availability

The original contributions presented in the study are included in the article/Supplementary Material, further inquiries can be directed to the corresponding authors.

## Abbreviations

Gal-3, galectin-3; Gal-3C, galectin-3C; Gal-7, galectin-7; Gal-9, galectin-9; Gal-9N, galectin-9N; Gal-9C, galectin-9C.
