# Supplementary material for: An Automated Approach to Assess Relative Galectin-Glycan Affinity Following Glycan Microarray Analysis
Source: Front Mol Biosci. 2022 Aug 11;9:893185. doi: 10.3389/fmolb.2022.893185 (PMC9403319; doi:10.3389/fmolb.2022.893185)
Supplement: Supplementary file 1 [file DataSheet2.PDF]

		hGal-3		hGal-3C		hGal-7		hGal-9		hGal-9N		hGal-9C	
Chart Number	Structure	K_D	% max	K_D	% max	K_D	% max	K_D	% max	K_D	% max	K_D	% max
1	Gala-Sp8												
2	Glca-Sp8												
3	Mana-Sp8												
4	GalNAca-Sp8												
5	GalNAca-Sp15												
6	Fuca-Sp8												
7	Fuca-Sp9												
8	Rhaa-Sp8												
9	Neu5Aca-Sp8												
10	Neu5Aca-Sp11												
11	Neu5Acb-Sp8												
12	Galb-Sp8												
13	Glcb-Sp8												
14	Manb-Sp8												
15	GalNAcb-Sp8												
16	GlcNAcb-Sp0												
17	GlcNAcb-Sp8												
18	GlcN(Gc)b-Sp8												
19	Galb1-4GlcNAcb1-6(Galb1-4GlcNAcb1-3)GalNAca-Sp8		11.6		8.2			0.8					
20	Galb1-4GlcNAcb1-6(Galb1-4GlcNAcb1-3)GalNAc-Sp14		6.5					2.5					
21	GlcNAcb1-6(GlcNAcb1-4)(GlcNAcb1-3)GlcNAc-Sp8												
22	6S(3S)Galb1-4(6S)GlcNAcb-Sp0												
23	6S(3S)Galb1-4GlcNAcb-Sp0												
24	(3S)Galb1-4(Fuca1-3)(6S)Glc-Sp0								13.7				
25	(3S)Galb1-4Glcb-Sp8	3.8					10.3	0.8					
26	(3S)Galb1-4(6S)Glcb-Sp0	2.0			11.5		22.1	0.7			14.2		
27	(3S)Galb1-4(6S)Glcb-Sp8	2.8			11.5		25.1	0.6			12.7		
28	(3S)Galb1-3(Fuca1-4)GlcNAcb-Sp8												
29	(3S)Galb1-3GalNAca-Sp8							0.5			20.5		
30	(3S)Galb1-3GlcNAcb-Sp0	2.4			21.2		43.1	0.6			6.4		10.1
31	(3S)Galb1-3GlcNAcb-Sp8	4.3			20.9		63.5	0.5					11.6
32	(3S)Galb1-4(Fuca1-3)GlcNAc-Sp0												
33	(3S)Galb1-4(Fuca1-3)GlcNAc-Sp8												
34	(3S)Galb1-4(6S)GlcNAcb-Sp0	2.2			21.8		84.2	0.5					14.0
35	(3S)Galb1-4(6S)GlcNAcb-Sp8	3.4			23.8		73.7	0.5					12.4
36	(3S)Galb1-4GlcNAcb-Sp0	3.7			23.4		40.6	1.1					
37	(3S)Galb1-4GlcNAcb-Sp8	1.9			20.2			0.8					
38	(3S)Galb-Sp8												
39	(6S)(4S)Galb1-4GlcNAcb-Sp0												
40	(4S)Galb1-4GlcNAcb-Sp8												
41	(6P)Mana-Sp8												
42	(6S)Galb1-4Glcb-Sp0												
43	(6S)Galb1-4Glcb-Sp8												
44	(6S)Galb1-4GlcNAcb-Sp8							3.1					
45	(6S)Galb1-4(6S)Glcb-Sp8												
46	Neu5Aca2-3(6S)Galb1-4GlcNAcb-Sp8												
47	(6S)GlcNAcb-Sp8												
48	Neu5,9Ac2a-Sp8												
49	Neu5,9Ac2a2-6Galb1-4GlcNAcb-Sp8												
50	Mana1-6(Mana1-3)Manb1-4GlcNAcb1-4GlcNAcb-Sp12												
51	Mana1-6(Mana1-3)Manb1-4GlcNAcb1-4GlcNAcb-Sp13												
52	GlcNAcb1-2Mana1-6(GlcNAcb1-2Mana1-3)Manb1-4GlcNAcb1-4GlcNAcb-Sp12												
53	GlcNAcb1-2Mana1-6(GlcNAcb1-2Mana1-3)Manb1-4GlcNAcb1-4GlcNAcb-Sp13								7.2				
54	Galb1-4GlcNAcb1-2Mana1-6(Galb1-4GlcNAcb1-2Mana1-3)Manb1-4GlcNAcb1-4GlcNAcb-Sp12		36.5		10.9			0.6					
55	Neu5Aca2-6Galb1-4GlcNAcb1-2Mana1-6(Neu5Aca2-6Galb1-4GlcNAcb1-2Mana1-3)Manb1-4GlcNAcb1-4GlcNAcb-Sp12								5.7				
56	Neu5Aca2-6Galb1-4GlcNAcb1-2Mana1-6(Neu5Aca2-6Galb1-4GlcNAcb1-2Man-a1-3)Manb1-4GlcNAcb1-4GlcNAcb-Sp21								26.4				
57	Neu5Aca2-6Galb1-4GlcNAcb1-2Mana1-6(Neu5Aca2-6Galb1-4GlcNAcb1-2Mana1-3)Manb1-4GlcNAcb1-4GlcNAcb-Sp24								38.4				
58	Fuca1-2Galb1-3GalNAcb1-3Gala-Sp9								9.6				
59	Fuca1-2Galb1-3GalNAcb1-3Gala1-4Galb1-4Glcb-Sp9												
60	Fuca1-2Galb1-3(Fuca1-4)GlcNAcb-Sp8												
61	Fuca1-2Galb1-3GalNAca-Sp8								6.5				

62	Fuca1-2Galb1-3GalNAca-Sp14								14.2				
63	Fuca1-2Galb1-3GalNAcb1-4(Neu5Aca2-3)Galb1-4Glcb-Sp0												
64	Fuca1-2Galb1-3GalNAcb1-4(Neu5Aca2-3)Galb1-4Glcb-Sp9												
65	Fuca1-2Galb1-3GlcNAcb1-3Galb1-4Glcb-Sp8		37.4		18.4		28.3	0.3		2.4			7.8
66	Fuca1-2Galb1-3GlcNAcb1-3Galb1-4Glcb-Sp10	7.4			25.6		58.0	0.3		2.2			12.0
67	Fuca1-2Galb1-3GlcNAcb-Sp0		19.1				21.0	1.0					
68	Fuca1-2Galb1-3GlcNAcb-Sp8		13.4				62.7	0.9					
69	Fuca1-2Galb1-4(Fuca1-3)GlcNAcb1-3Galb1-4(Fuca1-3)GlcNAcb-Sp0												
70	Fuca1-2Galb1-4(Fuca1-3)GlcNAcb1-3Galb1-4(Fuca1-3)GlcNAcb1-3Galb1-4(Fuca1-3)GlcNAcb-Sp0					2.0							
71	Fuca1-2Galb1-4(Fuca1-3)GlcNAcb-Sp0								9.4				
72	Fuca1-2Galb1-4(Fuca1-3)GlcNAcb-Sp8												
73	Fuca1-2Galb1-4GlcNAcb1-3Galb1-4GlcNAcb-Sp0		58.5		64.0	0.3		0.6					7.8
74	Fuca1-2Galb1-4GlcNAcb1-3Galb1-4GlcNAcb1-3Galb1-4GlcNAcb-Sp0	1.9		8.2									
75	Fuca1-2Galb1-4GlcNAcb-Sp0		15.3					1.8					
76	Fuca1-2Galb1-4GlcNAcb-Sp8		16.6					1.7					
77	Fuca1-2Galb1-4Glcb-Sp0		8.9					2.3					
78	Fuca1-2Galb-Sp8												
79	Fuca1-3GlcNAcb-Sp8												
80	Fuca1-4GlcNAcb-Sp8												
81	Fucb1-3GlcNAcb-Sp8												
82	GalNAca1-3(Fuca1-2)Galb1-3GlcNAcb-Sp0	6.7			26.1		7.7	0.3		1.9			10.1
83	GalNAca1-3(Fuca1-2)Galb1-4(Fuca1-3)GlcNAcb-Sp0												
84	(3S)Galb1-4(Fuca1-3)Glcb-Sp0								8.6				
85	GalNAca1-3(Fuca1-2)Galb1-4GlcNAcb-Sp0	1.7			46.2			0.3			36.0		33.3
86	GalNAca1-3(Fuca1-2)Galb1-4GlcNAcb-Sp8	2.2			52.5	0.1		0.3					47.5
87	GalNAca1-3(Fuca1-2)Galb1-4Glcb-Sp0		38.5		23.2			0.3		1.7			
88	GlcNAcb1-3Galb1-3GalNAca-Sp8							2.2					
89	GalNAca1-3(Fuca1-2)Galb-Sp8												
90	GalNAca1-3(Fuca1-2)Galb-Sp18												
91	GalNAca1-3GalNAcb-Sp8												
92	GalNAca1-3Galb-Sp8												
93	GalNAca1-4(Fuca1-2)Galb1-4GlcNAcb-Sp8		27.6		11.0				8.3				
94	GalNAcb1-3GalNAca-Sp8												
95	GalNAcb1-3(Fuca1-2)Galb-Sp8								39.8				
96	GalNAcb1-3Gala1-4Galb1-4GlcNAcb-Sp0								65.1				
97	GalNAcb1-4(Fuca1-3)GlcNAcb-Sp0												
98	GalNAcb1-4GlcNAcb-Sp0								82.1				
99	GalNAcb1-4GlcNAcb-Sp8								56.7				
100	Gala1-2Galb-Sp8												
101	Gala1-3(Fuca1-2)Galb1-3GlcNAcb-Sp0	2.1			67.0		14.8	0.4			6.7		47.7
102	Gala1-3(Fuca1-2)Galb1-3GlcNAcb-Sp8	1.5			57.2		28.3	0.3					60.3
103	Gala1-3(Fuca1-2)Galb1-4(Fuca1-3)GlcNAcb-Sp0												
104	Gala1-3(Fuca1-2)Galb1-4(Fuca1-3)GlcNAcb-Sp8												
105	Gala1-3(Fuca1-2)Galb1-4GlcNAc-Sp0	0.9		5.3				0.3					57.6
106	Gala1-3(Fuca1-2)Galb1-4Glcb-Sp0	2.4			54.4		59.5	0.3			9.5		
107	Gala1-3(Fuca1-2)Galb-Sp8						26.7		7.5				
108	Gala1-3(Fuca1-2)Galb-Sp18												
109	Gala1-4(Gala1-3)Galb1-4GlcNAcb-Sp8												
110	Gala1-3GalNAca-Sp8								7.9				
111	Gala1-3GalNAca-Sp16												
112	Gala1-3GalNAcb-Sp8												
113	Gala1-3Galb1-4(Fuca1-3)GlcNAcb-Sp8		25.1		10.9								
114	Gala1-3Galb1-3GlcNAcb-Sp0		73.4		30.6		7.1	0.4					
115	Gala1-3Galb1-4GlcNAcb-Sp8		13.7					0.7					
116	Gala1-3Galb1-4Glcb-Sp0		22.4		8.83			0.6					
117	Gala1-3Galb1-4Glc-Sp10						7.0	0.6					
118	Gala1-3Galb-Sp8												
119	Gala1-4(Fuca1-2)Galb1-4GlcNAcb-Sp8												
120	Gala1-4Galb1-4GlcNAcb-Sp0												
121	Gala1-4Galb1-4GlcNAcb-Sp8												
122	Gala1-4Galb1-4Glcb-Sp0												
123	Gala1-4GlcNAcb-Sp8												
124	Gala1-6Glcb-Sp8												
125	Galb1-2Galb-Sp8												
126	Galb1-3(Fuca1-4)GlcNAcb1-3Galb1-4(Fuca1-3)GlcNAcb-Sp0												
127	Galb1-3GlcNAcb1-3Galb1-4(Fuca1-3)GlcNAcb-Sp0		7.7				25.0	1.2					
128	Galb1-3(Fuca1-4)GlcNAc-Sp0												
129	Galb1-3(Fuca1-4)GlcNAc-Sp8												
130	Fuca1-4(Galb1-3)GlcNAcb-Sp8												
131	Galb1-4GlcNAcb1-6GalNAca-Sp8								70.3				

132	Galb1-4GlcNAcb1-6GalNAc-Sp14								5.5				
133	GlcNAcb1-6(Galb1-3)GalNAca-Sp8							3.1					
134	Neu5Aca2-6(Galb1-3)GalNAca-Sp8								7.1				
135	Neu5Aca2-6(Galb1-3)GalNAca-Sp14												
136	Neu5Acb2-6(Galb1-3)GalNAca-Sp8												
137	Neu5Aca2-6(Galb1-3)GlcNAcb1-4Galb1-4Glcb-Sp10							2.6					
138	Galb1-3GalNAca-Sp8								82.9				
139	Galb1-3GalNAca-Sp14								25.2				
140	Galb1-3GalNAca-Sp16								17.5				
141	Galb1-3GalNAcb-Sp8							2.8					
142	Galb1-3GalNAcb1-3Gala1-4Galb1-4Glcb-Sp0							0.7					
143	Galb1-3GalNAcb1-4(Neu5Aca2-3)Galb1-4Glcb-Sp0							3.1					
144	Galb1-3GalNAcb1-4Galb1-4Glcb-Sp8							1.9					
145	Galb1-3Galb-Sp8							2.1					
146	Galb1-3GlcNAcb1-3Galb1-4GlcNAcb-Sp0		36.1		21.5		8.9	0.4					14.7
147	Galb1-3GlcNAcb1-3Galb1-4Glcb-Sp10	7.7			15.0		24.3	0.4		3.1			9.4
148	Galb1-3GlcNAcb-Sp0		10.7					1.4					
149	Galb1-3GlcNAcb-Sp8						7.5	1.7					
150	Galb1-4(Fuca1-3)GlcNAcb-Sp0												
151	Galb1-4(Fuca1-3)GlcNAcb-Sp8												
152	Galb1-4(Fuca1-3)GlcNAcb1-3Galb1-4(Fuca1-3)GlcNAcb-Sp0												
153	Galb1-4(Fuca1-3)GlcNAcb1-3Galb1-4(Fuca1-3)GlcNAcb1-3Galb1-4(Fuca1-3)GlcNAcb-Sp0								20.6				
154	Galb1-4(6S)Glcb-Sp0						7.2	2.2					
155	Galb1-4(6S)Glcb-Sp8						8.2	2.4					
156	Galb1-4GalNAca1-3(Fuca1-2)Galb1-4GlcNAcb-Sp8	2.1			74.1			0.3			56.4		40.2
157	Galb1-4GalNAcb1-3(Fuca1-2)Galb1-4GlcNAcb-Sp8							2.9					
158	Galb1-4GlcNAcb1-3GalNAca-Sp8							1.5					
159	Galb1-4GlcNAcb1-3GalNAc-Sp14		6.6					2.8					
160	Galb1-4GlcNAcb1-3Galb1-4(Fuca1-3)GlcNAcb1-3Galb1-4(Fuca1-3)GlcNAcb-Sp0							1.1					
161	Galb1-4GlcNAcb1-3Galb1-4GlcNAcb1-3Galb1-4GlcNAcb-Sp0	6.2			56.0		6.6	0.3			53.0		67.4
162	Galb1-4GlcNAcb1-3Galb1-4GlcNAcb-Sp0	7.0			70.5		13.9	0.3			19.5		27.6
163	Galb1-4GlcNAcb1-3Galb1-4Glcb-Sp0	7.3			29.7		29.5	0.3		2.2			
164	Galb1-4GlcNAcb1-3Galb1-4Glcb-Sp8		50.8		28.6		62.3	0.3		1.9			15.2
165	Galb1-4GlcNAcb1-6(Galb1-3)GalNAca-Sp8							1.4					
166	Galb1-4GlcNAcb1-6(Galb1-3)GalNAc-Sp14							2.0					
167	Galb1-4GlcNAcb-Sp0								52.7				
168	Galb1-4GlcNAcb-Sp8							2.0					
169	Galb1-4GlcNAcb-Sp23								43.3				
170	Galb1-4Glcb-Sp0								11.7				
171	Galb1-4Glcb-Sp8								20.7				
172	GlcNAca1-3Galb1-4GlcNAcb-Sp8		7.9					1.3					
173	GlcNAca1-6Galb1-4GlcNAcb-Sp8												
174	GlcNAcb1-2Galb1-3GalNAca-Sp8							3.2					
175	GlcNAcb1-6(GlcNAcb1-3)GalNAca-Sp8												
176	GlcNAcb1-6(GlcNAcb1-3)GalNAca-Sp14												
177	GlcNAcb1-6(GlcNAcb1-3)Galb1-4GlcNAcb-Sp8								9.5				
178	GlcNAcb1-3GalNAca-Sp8								14.2				
179	GlcNAcb1-3GalNAca-Sp14												
180	GlcNAcb1-3Galb-Sp8												
181	GlcNAcb1-3Galb1-4GlcNAcb-Sp0		16.6		11.6			0.6					
182	GlcNAcb1-3Galb1-4GlcNAcb-Sp8		35.2		22.1			0.6					
183	GlcNAcb1-3Galb1-4GlcNAcb1-3Galb1-4GlcNAcb-Sp0	3.8			73.9			0.3			10.0		52.8
184	GlcNAcb1-3Galb1-4Glcb-Sp0		13.6					0.7			9.5		
185	GlcNAcb1-4-MDPLys												
186	GlcNAcb1-6(GlcNAcb1-4)GalNAca-Sp8												
187	GlcNAcb1-4Galb1-4GlcNAcb-Sp8												
188	GlcNAcb1-4GlcNAcb1-4GlcNAcb1-4GlcNAcb1-4GlcNAcb1-4GlcNAcb1-Sp8												
189	GlcNAcb1-4GlcNAcb1-4GlcNAcb1-4GlcNAcb1-4GlcNAcb1-Sp8												
190	GlcNAcb1-4GlcNAcb1-4GlcNAcb-Sp8												
191	GlcNAcb1-6GalNAca-Sp8								11.9				
192	GlcNAcb1-6GalNAca-Sp14												
193	GlcNAcb1-6Galb1-4GlcNAcb-Sp8								10.7				
194	Glca1-4Glcb-Sp8								7.2				
195	Glca1-4Glca-Sp8												
196	Glca1-6Glca1-6Glcb-Sp8												
197	Glcb1-4Glcb-Sp8												
198	Glcb1-6Glcb-Sp8												
199	G-ol-Sp8												
200	GlcAa-Sp8												

201	GlcAb-Sp8												
202	GlcAb1-3Galb-Sp8												
203	GlcAb1-6Galb-Sp8												
204	KDNa2-3Galb1-3GlcNAcb-Sp0		14.1				5.7		50.0				
205	KDNa2-3Galb1-4GlcNAcb-Sp0		7.4						6.0				
206	Mana1-2Mana1-2Mana1-3Mana-Sp9												
207	Mana1-2Mana1-6(Mana1-2Mana1-3)Mana-Sp9												
208	Mana1-2Mana1-3Mana-Sp9												
209	Mana1-6(Mana1-3)Mana-Sp9												
210	Mana1-2Mana1-2Mana1-6(Mana1-3)Mana-Sp9												
211	Mana1-6(Mana1-3)Mana1-6(Mana1-2Mana1-3)Manb1-4GlcNAcb1-4GlcNAcb-Sp12						7.0		41.4				13.3
212	Mana1-6(Mana1-3)Mana1-6(Mana1-3)Manb1-4GlcNAcb1-4GlcNAcb-Sp12												
213	Manb1-4GlcNAcb-Sp0												
214	Neu5Aca2-3Galb1-4GlcNAcb1-3Galb1-4(Fuca1-3)GlcNAcb-Sp0												
215	(3S)Galb1-4(Fuca1-3)(6S)GlcNAcb-Sp8							1.5					
216	Fuca1-2(6S)Galb1-4GlcNAcb-Sp0												
217	Fuca1-2Galb1-4(6S)GlcNAcb-Sp8		16.7		7.3			1.7					
218	Fuca1-2(6S)Galb1-4(6S)Glcb-Sp0												
219	Neu5Aca2-3Galb1-3GalNAca-Sp8								50.2				
220	Neu5Aca2-3Galb1-3GalNAca-Sp14								39.0				
221	GalNAcb1-4(Neu5Aca2-8Neu5Aca2-8Neu5Aca2-8Neu5Aca2-3)Galb1-4Glcb-Sp0												
222	GalNAcb1-4(Neu5Aca2-8Neu5Aca2-8Neu5Aca2-3)Galb1-4Glcb-Sp0												
223	Neu5Aca2-8Neu5Aca2-8Neu5Aca2-3Galb1-4Glcb-Sp0						12.5		12.5				
224	GalNAcb1-4(Neu5Aca2-8Neu5Aca2-3)Galb1-4Glcb-Sp0												
225	Neu5Aca2-8Neu5Aca2-8Neu5Aca-Sp8												
226	GalNAcb1-4(Neu5Aca2-3)Galb1-4GlcNAcb-Sp0												
227	GalNAcb1-4(Neu5Aca2-3)Galb1-4GlcNAcb-Sp8					1.7		2.7			8.8		83.1
228	GalNAcb1-4(Neu5Aca2-3)Galb1-4Glcb-Sp0												
229	Neu5Aca2-3Galb1-3GalNAcb1-4(Neu5Aca2-3)Galb1-4Glcb-Sp0								56.5				
230	Neu5Aca2-6(Neu5Aca2-3)GalNAca-Sp8												
231	Neu5Aca2-3GalNAca-Sp8								12.0				
232	Neu5Aca2-3GalNAcb1-4GlcNAcb-Sp0		8.0						9.0				
233	Neu5Aca2-3Galb1-3(6S)GlcNAc-Sp8								34.1				
234	Neu5Aca2-3Galb1-3(Fuca1-4)GlcNAcb-Sp8												
235	Neu5Aca2-3Galb1-3(Fuca1-4)GlcNAcb1-3Galb1-4(Fuca1-3)GlcNAcb-Sp0												
236	Neu5Aca2-3Galb1-4(Neu5Aca2-3Galb1-3)GlcNAcb-Sp8												
237	Neu5Aca2-3Galb1-3(6S)GalNAca-Sp8							2.3					
238	Neu5Aca2-6(Neu5Aca2-3Galb1-3)GalNAca-Sp8												
239	Neu5Aca2-6(Neu5Aca2-3Galb1-3)GalNAca-Sp14												
240	Neu5Aca2-3Galb-Sp8												
241	Neu5Aca2-3Galb1-3GalNAcb1-3Gala1-4Galb1-4Glcb-Sp0							0.7					
242	Neu5Aca2-3Galb1-3GlcNAcb1-3Galb1-4GlcNAcb-Sp0	5.8			28.7		34.0	0.3					9.2
243	Fuca1-2(6S)Galb1-4Glcb-Sp0												
244	Neu5Aca2-3Galb1-3GlcNAcb-Sp0	8.0					16.9		57.7				
245	Neu5Aca2-3Galb1-4(6S)GlcNAcb-Sp8	7.1			15.9		6.9		49.2				
246	Neu5Aca2-3Galb1-4(Fuca1-3)(6S)GlcNAcb-Sp8												
247	Neu5Aca2-3Galb1-4(Fuca1-3)GlcNAcb1-3Galb1-4(Fuca1-3)GlcNAcb1-3Galb1-4(Fuca1-3)GlcNAcb-Sp0												
248	Neu5Aca2-3Galb1-4(Fuca1-3)GlcNAcb-Sp0												
249	Neu5Aca2-3Galb1-4(Fuca1-3)GlcNAcb-Sp8												
250	Neu5Aca2-3Galb1-4(Fuca1-3)GlcNAcb1-3Galb-Sp8												
251	Neu5Aca2-3Galb1-4(Fuca1-3)GlcNAcb1-3Galb1-4GlcNAcb-Sp8								10.2				
252	Neu5Aca2-3Galb1-4GlcNAcb1-3Galb1-4GlcNAcb1-3Galb1-4GlcNAcb-Sp0						23.0	0.4					44.0
253	Neu5Aca2-3Galb1-4GlcNAcb-Sp0		6.7				5.9		8.8				
254	Neu5Aca2-3Galb1-4GlcNAcb-Sp8		9.7						13.8				
255	Neu5Aca2-3Galb1-4GlcNAcb1-3Galb1-4GlcNAcb-Sp0		20.9		12.2				77.6				
256	Fuca1-2Galb1-4(6S)Glcb-Sp0		12.4				40.9	2.3					
257	Neu5Aca2-3Galb1-4Glcb-Sp0								8.9				
258	Neu5Aca2-3Galb1-4Glcb-Sp8						9.9		14.4				
259	Neu5Aca2-6GalNAca-Sp8												
260	Neu5Aca2-6GalNAcb1-4GlcNAcb-Sp0												

261	Neu5Aca2-6Galb1-4(6S)GlcNAcb-Sp8												
262	Neu5Aca2-6Galb1-4GlcNAcb-Sp8												
263	Neu5Aca2-6Galb1-4GlcNAcb1-3Galb1-4(Fuca1-3)GlcNAcb1-3Galb1-4(Fuca1-3)GlcNAcb-Sp0								26.6				
264	Neu5Aca2-6Galb1-4GlcNAcb1-3Galb1-4GlcNAcb-Sp0		25.5		27.3		51.7	0.4			54.1		21.1
265	Neu5Aca2-6Galb1-4Glcb-Sp0								13.7				
266	Neu5Aca2-6Galb1-4Glcb-Sp8												
267	Neu5Aca2-6Galb-Sp8												
268	Neu5Aca2-8Neu5Aca-Sp8												
269	Neu5Aca2-8Neu5Aca2-3Galb1-4Glcb-Sp0								11.6				
270	Galb1-3(Fuca1-4)GlcNAcb1-3Galb1-3(Fuca1-4)GlcNAcb-Sp0												
271	Neu5Acb2-6GalNAca-Sp8												
272	Neu5Acb2-6Galb1-4GlcNAcb-Sp8												
273	Neu5Gca2-3Galb1-3(Fuca1-4)GlcNAcb-Sp0												
274	Neu5Gca2-3Galb1-3GlcNAcb-Sp0		18.8					3.2					
275	Neu5Gca2-3Galb1-4(Fuca1-3)GlcNAcb-Sp0												
276	Neu5Gca2-3Galb1-4GlcNAcb-Sp0		11.2						17.2				
277	Neu5Gca2-3Galb1-4Glcb-Sp0						5.3		21.2				
278	Neu5Gca2-6GalNAca-Sp0												
279	Neu5Gca2-6Galb1-4GlcNAcb-Sp0												
280	Neu5Gca-Sp8												
281	Neu5Aca2-3Galb1-4GlcNAcb1-6(Galb1-3)GalNAca-Sp14								62.4				
282	Galb1-3GlcNAcb1-3Galb1-3GlcNAcb-Sp0		45.8		24.7		73.4	0.2			60.5		33.3
283	Galb1-4(Fuca1-3)(6S)GlcNAcb-Sp0								7.5				
284	Galb1-4(Fuca1-3)(6S)Glcb-Sp0												
285	Galb1-4(Fuca1-3)GlcNAcb1-3Galb1-3(Fuca1-4)GlcNAcb-Sp0												
286	Galb1-4GlcNAcb1-3Galb1-3GlcNAcb-Sp0		30.7		20.7		9.2	0.3			68.8		26.9
287	Neu5Aca2-3Galb1-3GlcNAcb1-3Galb1-3GlcNAcb-Sp0	5.9			28.6		54.5	0.3			50.0		24.5
288	Neu5Aca2-3Galb1-4GlcNAcb1-3Galb1-3GlcNAcb-Sp0		26.0		11.7			1.6					
289	4S(3S)Galb1-4GlcNAcb-Sp0												
290	(6S)Galb1-4(6S)GlcNAcb-Sp0								7.5				
291	(6P)Glcb-Sp10												
292	Galb1-3Galb1-4GlcNAcb-Sp8						22.4	0.3					26.2
293	Neu5Aca2-6Galb1-4GlcNAcb1-2Mana1-6(Galb1-4GlcNAcb1-2Mana1-3)Manb1-4GlcNAcb1-4GlcNAcb-Sp12							2.7					
294	Galb1-4GlcNAcb1-6(Galb1-4GlcNAcb1-3)Galb1-4GlcNAc-Sp0		17.5		16.5		11.3	0.9					
295	GlcNAcb1-6(Galb1-4GlcNAcb1-3)Galb1-4GlcNAc-Sp0		9.2					1.3					
296	Galb1-4GlcNAca1-6Galb1-4GlcNAcb-Sp0		11.1					1.6					
297	Galb1-4GlcNAcb1-6Galb1-4GlcNAcb-Sp0							2.4					
298	GalNAcb1-3Galb-Sp8							1.5					
299	GlcAb1-3GlcNAcb-Sp8												
300	Neu5Aca2-6Galb1-4GlcNAcb1-2Mana1-6(GlcNAcb1-2Mana1-3)Manb1-4GlcNAcb1-4GlcNAcb-Sp12												
301	GlcNAcb1-3Man-Sp10												
302	GlcNAcb1-4GlcNAcb-Sp10												
303	GlcNAcb1-4GlcNAcb-Sp12												
304	MurNAcb1-4GlcNAcb-Sp10												
305	Mana1-6Manb-Sp10												
306	Mana1-6(Mana1-3)Mana1-6(Mana1-3)Manb-Sp10												
307	Mana1-2Mana1-6(Mana1-3)Mana1-6(Mana1-2Mana1-2Mana1-3)Mana-Sp9												
308	Mana1-2Mana1-6(Mana1-2Mana1-3)Mana1-6(Mana1-2Mana1-2Mana1-3)Mana-Sp9						17.7		6.5				
309	Neu5Aca2-3Galb1-4GlcNAcb1-6(Neu5Aca2-3Galb1-3)GalNAca-Sp14		7.1						40.5				
310	Neu5Aca2-6Galb1-4GlcNAcb1-2Mana1-6(Neu5Aca2-3Galb1-4GlcNAcb1-2Mana1-3)Manb1-4GlcNAcb1-4GlcNAcb-Sp12		14.0						61.7				
311	Galb1-4GlcNAcb1-2Mana1-6(Neu5Aca2-6Galb1-4GlcNAcb1-2Mana1-3)Manb1-4GlcNAcb1-4GlcNAcb-Sp12								61.3				
312	Neu5Aca2-8Neu5Aca2-8Neu5Acb-Sp8												
313	Neu5Gcb2-6Galb1-4GlcNAc-Sp8												
314	Galb1-3GlcNAcb1-2Mana1-6(Galb1-3GlcNAcb1-2Mana1-3)Manb1-4GlcNAcb1-4GlcNAcb-Sp19		6.3				62.2	0.4					
315	Neu5Aca2-3Galb1-4GlcNAcb1-2Mana1-6(Neu5Aca2-3Galb1-4GlcNAcb1-2Mana1-3)Manb1-4GlcNAcb1-4GlcNAcb-Sp12	8.4			24.8		17.2		91.0				

316	Neu5Aca2-3Galb1-4GlcNAcb1-2Mana1-6(Neu5Aca2-6Galb1-4GlcNAcb1-2Mana1-3)Manb1-4GlcNAcb1-4GlcNAcb-Sp12		13.6					2.4					
317	Galb1-4(Fuca1-3)GlcNAcb1-2Mana1-6(Galb1-4(Fuca1-3)GlcNAcb1-2Mana1-3)Manb1-4GlcNAcb1-4GlcNAcb-Sp20												
318	Neu5,9Ac2a2-3Galb1-3GlcNAcb-Sp0		8.9				9.8		50.7				
319	Neu5Aca2-6Galb1-4GlcNAcb1-3Galb1-3GlcNAcb-Sp0	4.6			40.5		93.2	0.3		0.4			48.2
320	Neu5Aca2-3Galb1-3(Fuca1-4)GlcNAcb1-3Galb1-3(Fuca1-4)GlcNAcb-Sp0												
321	Neu5Aca2-6Galb1-4GlcNAcb1-3Galb1-4GlcNAcb1-3Galb1-4GlcNAcb-Sp0	2.5			68.4		75.5	0.3		1.7			52.8
322	Gala1-4Galb1-4GlcNAcb1-3Galb1-4Glcb-Sp0		44.7		29.5		32.0	0.3		3.2			
323	GalNAcb1-3Gala1-4Galb1-4GlcNAcb1-3Galb1-4Glcb-Sp0		49.1		39.1			0.3			76.2		
324	GalNAca1-3(Fuca1-2)Galb1-4GlcNAcb1-3Galb1-4GlcNAcb-Sp0	1.7		8.1			73.4	0.2			78.1		61.9
325	GalNAca1-3(Fuca1-2)Galb1-4GlcNAcb1-3Galb1-4GlcNAcb1-3Galb1-4GlcNAcb-Sp0	2.1			62.5		11.1	0.2			37.1		45.0
326	Neu5Aca2-3Galb1-4(Fuca1-3)GlcNAcb1-6(Neu5Aca2-3Galb1-3)GalNAc-Sp14								11.4				
327	GlcNAca1-4Galb1-4GlcNAcb1-3Galb1-4GlcNAcb1-3Galb1-4GlcNAcb-Sp0	5.1			70.9			0.3			17.4		67.3
328	GlcNAca1-4Galb1-4GlcNAcb-Sp0												
329	GlcNAca1-4Galb1-3GlcNAcb-Sp0												
330	GlcNAca1-4Galb1-4GlcNAcb1-3Galb1-4Glcb-Sp0		31.1		16.0			0.4			45.5		
331	GlcNAca1-4Galb1-4GlcNAcb1-3Galb1-4(Fuca1-3)GlcNAcb1-3Galb1-4(Fuca1-3)GlcNAcb-Sp0								6.8				
332	GlcNAca1-4Galb1-4GlcNAcb1-3Galb1-4GlcNAcb-Sp0	8.0			41.1			0.3					21.5
333	GlcNAca1-4Galb1-3GalNAc-Sp14												
334	Neu5Aca2-6Galb1-4GlcNAcb1-2Mana1-6(Mana1-3)Manb1-4GlcNAcb1-4GlcNAc-Sp12												
335	Mana1-6(Neu5Aca2-6Galb1-4GlcNAcb1-2Mana1-3)Manb1-4GlcNAcb1-4GlcNAc-Sp12								16.0				
336	Neu5Aca2-6Galb1-4GlcNAcb1-2Mana1-6Manb1-4GlcNAcb1-4GlcNAc-Sp12												
337	Neu5Aca2-6Galb1-4GlcNAcb1-2Mana1-3Manb1-4GlcNAcb1-4GlcNAc-Sp12												
338	Galb1-4GlcNAcb1-2Mana1-3Manb1-4GlcNAcb1-4GlcNAc-Sp12		8.7					1.4					
339	Galb1-4GlcNAcb1-2Mana1-6Manb1-4GlcNAcb1-4GlcNAc-Sp12		8.5					2.6					
340	Mana1-6(Galb1-4GlcNAcb1-2Mana1-3)Manb1-4GlcNAcb1-4GlcNAcb-Sp12		7.0					1.6					
341	GlcNAcb1-2Mana1-6(GlcNAcb1-2Mana1-3)Manb1-4GlcNAcb1-4(Fuca1-6)GlcNAcb-Sp22												
342	Galb1-4GlcNAcb1-2Mana1-6(Galb1-4GlcNAcb1-2Mana1-3)Manb1-4GlcNAcb1-4(Fuca1-6)GlcNAcb-Sp22		12.7				38.9	1.1					
343	Galb1-3GlcNAcb1-2Mana1-6(Galb1-3GlcNAcb1-2Mana1-3)Manb1-4GlcNAcb1-4(Fuca1-6)GlcNAcb-Sp22						66.1	1.0					
344	(6S)GlcNAcb1-3Galb1-4GlcNAcb-Sp0								66.3				
345	KDNa2-3Galb1-4(Fuca1-3)GlcNAc-Sp0												
346	KDNa2-6Galb1-4GlcNAc-Sp0								5.7				
347	KDNa2-3Galb1-4Glc-Sp0												
348	KDNa2-3Galb1-3GalNAca-Sp14								11.7				
349	Fuca1-2Galb1-3GlcNAcb1-2Mana1-6(Fuca1-2Galb1-3GlcNAcb1-2Mana1-3)Manb1-4GlcNAcb1-4GlcNAcb-Sp20		6.5		16.4		67.7	1.4			15.6		16.8
350	Fuca1-2Galb1-4GlcNAcb1-2Mana1-6(Fuca1-2Galb1-4GlcNAcb1-2Mana1-3)Manb1-4GlcNAcb1-4GlcNAcb-Sp20		36.8		19.0			0.9			11.1		30.0
351	Fuca1-2Galb1-4(Fuca1-3)GlcNAcb1-2Mana1-6(Fuca1-2Galb1-4(Fuca1-3)GlcNAcb1-2Mana1-3)Manb1-4GlcNAcb1-4GlcNAb-Sp20							3.1					
352	Gala1-3Galb1-4GlcNAcb1-2Mana1-6(Gala1-3Galb1-4GlcNAcb1-2Mana1-3)Manb1-4GlcNAcb1-4GlcNAcb-Sp20	2.2			100.0		72.4	0.2					47.8
353	Galb1-4GlcNAcb1-2Mana1-6(Mana1-3)Manb1-4GlcNAcb1-4GlcNAcb-Sp12							2.7					
354	Fuca1-4(Galb1-3)GlcNAcb1-2Mana1-6(Fuca1-4(Galb1-3)GlcNAcb1-2Mana1-3)Manb1-4GlcNAcb1-4(Fuca1-6)GlcNAcb-Sp22										6.7		11.8
355	Neu5Aca2-6GlcNAcb1-4GlcNAc-Sp21												
356	Neu5Aca2-6GlcNAcb1-4GlcNAcb1-4GlcNAc-Sp21												

357	Galb1-4(Fuca1-3)GlcNAcb1-6(Fuca1-2Galb1-4GlcNAcb1-3)Galb1-4Glc-Sp21	10.0					31.3	0.7					
358	Galb1-4GlcNAcb1-2Mana1-6(Galb1-4GlcNAcb1-4(Galb1-4GlcNAcb1-2)Mana1-3)Manb1-4GlcNAcb1-4GlcNAc-Sp21		60.3		53.9		76.1	0.3					15.8
359	GalNAca1-3(Fuca1-2)Galb1-4GlcNAcb1-2Mana1-6(GalNAca1-3(Fuca1-2)Galb1-4GlcNAcb1-2Mana1-3)Manb1-4GlcNAcb1-4GlcNAcb-Sp20	1.0		4.8				0.2		1.7			50.7
360	Gala1-3(Fuca1-2)Galb1-4GlcNAcb1-2Mana1-6(Gala1-3(Fuca1-2)Galb1-4GlcNAcb1-2Mana1-3)Manb1-4GlcNAcb1-4GlcNAcb-Sp20	0.5		1.7			79.9	0.2			21.8	2.2	
361	Gala1-3Galb1-4(Fuca1-3)GlcNAcb1-2Mana1-6(Gala1-3Galb1-4(Fuca1-3)GlcNAcb1-2Mana1-3)Manb1-4GlcNAcb1-4GlcNAcb-Sp20							2.9					
362	GalNAca1-3(Fuca1-2)Galb1-3GlcNAcb1-2Mana1-6(GalNAca1-3(Fuca1-2)Galb1-3GlcNAcb1-2Mana1-3)Manb1-4GlcNAcb1-4GlcNAcb-Sp20	2.8			81.7			0.3		0.3			10.7
363	Gala1-3(Fuca1-2)Galb1-3GlcNAcb1-2Mana1-6(Gala1-3(Fuca1-2)Galb1-3GlcNAcb1-2Mana1-3)Manb1-4GlcNAcb1-4GlcNAcb-Sp20	1.7		4.9			74.4	0.3		1.7		3.1	
364	Fuca1-4(Fuca1-2Galb1-3)GlcNAcb1-2Mana1-3(Fuca1-4(Fuca1-2Galb1-3)GlcNAcb1-2Mana1-3)Manb1-4GlcNAcb1-4GlcNAcb-Sp19												
365	Neu5Aca2-3Galb1-4GlcNAcb1-3GalNAc-Sp14		7.3						9.4				
366	Neu5Aca2-6Galb1-4GlcNAcb1-3GalNAc-Sp14												
367	Neu5Aca2-3Galb1-4(Fuca1-3)GlcNAcb1-3GalNAca-Sp14								12.6				
368	GalNAcb1-4GlcNAcb1-2Mana1-6(GalNAcb1-4GlcNAcb1-2Mana1-3)Manb1-4GlcNAcb1-4GlcNAc-Sp12							2.4			6.7		11.0
369	Galb1-3GalNAca1-3(Fuca1-2)Galb1-4Glc-Sp0		33.4		17.8		33.0	0.3		2.4			
370	Galb1-3GalNAca1-3(Fuca1-2)Galb1-4GlcNAc-Sp0	2.2			50.8		8.4	0.3			15.9		42.7
371	Galb1-3GlcNAcb1-3Galb1-4GlcNAcb1-6(Galb1-3GlcNAcb1-3)Galb1-4Glcb-Sp21		56.2		38.0		87.1	0.3					39.7
372	Galb1-4(Fuca1-3)GlcNAcb1-6(Galb1-3GlcNAcb1-3)Galb1-4Glc-Sp21						63.4	1.7					
373	Galb1-4GlcNAcb1-6(Fuca1-4(Fuca1-2Galb1-3)GlcNAcb1-3)Galb1-4Glc-Sp21							2.0					
374	Galb1-4(Fuca1-3)GlcNAcb1-6(Fuca1-4(Fuca1-2Galb1-3)GlcNAcb1-3)Galb1-4Glc-Sp21							2.1					
375	Galb1-3GlcNAcb1-3Galb1-4(Fuca1-3)GlcNAcb1-6(Galb1-3GlcNAcb1-3)Galb1-4Glc-Sp21						38.4	0.4					
376	Galb1-4GlcNAcb1-6(Galb1-4GlcNAcb1-2)Mana1-6(Galb1-4GlcNAcb1-4(Galb1-4GlcNAcb1-2)Mana1-3)Manb1-4GlcNAcb1-4GlcNAcb-Sp21		84.4		72.2			0.3					11.3
377	GlcNAcb1-2Mana1-6(GlcNAcb1-4(GlcNAcb1-2)Mana1-3)Manb1-4GlcNAcb1-4GlcNAc-Sp21								7.5				
378	Fuca1-2Galb1-3GalNAca1-3(Fuca1-2)Galb1-4Glcb-Sp0							0.9					
379	Fuca1-2Galb1-3GalNAca1-3(Fuca1-2)Galb1-4GlcNAcb-Sp0		17.9		12.5			0.4					14.3
380	Galb1-3GlcNAcb1-3GalNAca-Sp14	6.3					5.0	3.0					
381	GalNAca1-3(Fuca1-2)Galb1-3GalNAca1-3(Fuca1-2)Galb1-4GlcNAcb-Sp0	9.2			10.9			0.3					9.8
382	Gala1-3Galb1-3GlcNAcb1-2Mana1-6(Gala1-3Galb1-3GlcNAcb1-2Mana1-3)Manb1-4GlcNAcb1-4GlcNAc-Sp19								49.9				
383	Gala1-3Galb1-3(Fuca1-4)GlcNAcb1-2Mana1-6(Gala1-3Galb1-3(Fuca1-4)GlcNAcb1-2Mana1-3)Manb1-4GlcNAcb1-4GlcNAc-Sp19										5.9		8.6
384	GlcNAcb1-2Mana1-6(Galb1-4GlcNAcb1-2Mana1-3)Manb1-4GlcNAcb1-4GlcNAc-Sp12		9.3					1.8					
385	Galb1-4GlcNAcb1-2Mana1-6(GlcNAcb1-2Mana1-3)Manb1-4GlcNAcb1-4GlcNAc-Sp12							2.3					
386	Neu5Aca2-3Galb1-3GlcNAcb1-3GalNAca-Sp14	2.6						1.9					
387	Fuca1-2Galb1-4GlcNAcb1-3GalNAca-Sp14		12.5						28.7				
388	Galb1-4(Fuca1-3)GlcNAcb1-3GalNAca-Sp14												
389	GalNAca1-3GalNAcb1-3Gala1-4Galb1-4GlcNAcb-Sp0							0.3		0.5			
390	Gala1-4Galb1-3GlcNAcb1-2Mana1-6(Gala1-4Galb1-3GlcNAcb1-2Mana1-3)Manb1-4GlcNAcb1-4GlcNAcb-Sp19												
391	Gala1-4Galb1-4GlcNAcb1-2Mana1-6(Gala1-4Galb1-4GlcNAcb1-2Mana1-3)Manb1-4GlcNAcb1-4GlcNAcb-Sp24								10.9				
392	Gala1-3Galb1-4GlcNAcb1-3GalNAca-Sp14		41.3		19.9								
393	Galb1-3GlcNAcb1-6Galb1-4GlcNAcb-Sp0						72.9	2.2					
394	Galb1-3GlcNAca1-6Galb1-4GlcNAcb-Sp0						37.0		49.9				

395	GalNAcb1-3Gala1-6Galb1-4Glcb-Sp8							2.8					
396	Gala1-3(Fuca1-2)Galb1-4(Fuca1-3)Glcb-Sp21												
397	Galb1-4GlcNAcb1-6(Neu5Aca2-6Galb1-3GlcNAcb1-3)Galb1-4Glc-Sp21							2.7					
398	Galb1-3GalNAcb1-4(Neu5Aca2-8Neu5Aca2-3)Galb1-4Glcb-Sp0								13.6				
399	Neu5Aca2-3Galb1-3GalNAcb1-4(Neu5Aca2-8Neu5Aca2-3)Galb1-4Glcb-Sp0												
400	Gala1-3(Fuca1-2)Galb1-4GlcNAcb1-3GalNAca-Sp14	1.2			59.4			0.3					28.1
401	GalNAca1-3(Fuca1-2)Galb1-4GlcNAcb1-3GalNAca-Sp14	3.4			23.3			0.4					
402	GalNAca1-3GalNAcb1-3Gala1-4Galb1-4Glcb-Sp0							0.3		0.4			
403	Fuca1-2Galb1-4(Fuca1-3)GlcNAcb1-3GalNAca-Sp14								16.1				
404	Gala1-3(Fuca1-2)Galb1-4(Fuca1-3)GlcNAcb1-3GalNAc-Sp14								8.5				
405	GalNAca1-3(Fuca1-2)Galb1-4(Fuca1-3)GlcNAcb1-3GalNAc-Sp14								14.2				
406	Galb1-4(Fuca1-3)GlcNAcb1-2Mana1-6(Galb1-4(Fuca1-3)GlcNAcb1-2Mana1-3)Manb1-4GlcNAcb1-4(Fuca1-6)GlcNAcb-Sp22								11.7				
407	Fuca1-2Galb1-4GlcNAcb1-2Mana1-6(Fuca1-2Galb1-4GlcNAcb1-2Mana1-3)Manb1-4GlcNAcb1-4(Fuca1-6)GlcNAcb-Sp22		10.4					2.1			11.3		29.4
408	GlcNAcb1-2(GlcNAcb1-6)Mana1-6(GlcNAcb1-2Mana1-3)Manb1-4GlcNAcb1-4GlcNAcb-Sp19												
409	Fuca1-2Galb1-3GlcNAcb1-3GalNAc-Sp14		21.4		11.2		12.1	1.4					
410	Gala1-3(Fuca1-2)Galb1-3GlcNAcb1-3GalNAc-Sp14	2.0			39.4		23.3	0.4					6.4
411	GalNAca1-3(Fuca1-2)Galb1-3GlcNAcb1-3GalNAc-Sp14		10.1					0.3			8.7		
412	Gala1-3Galb1-3GlcNAcb1-3GalNAc-Sp14		23.5		9.6		10.6	1.1					
413	Fuca1-2Galb1-3GlcNAcb1-2Mana1-6(Fuca1-2Galb1-3GlcNAcb1-2Mana1-3)Manb1-4GlcNAcb1-4(Fuca1-6)GlcNAcb-Sp22							2.8			7.6		16.6
414	Gala1-3(Fuca1-2)Galb1-4GlcNAcb1-2Mana1-6(Gala1-3(Fuca1-2)Galb1-4GlcNAcb1-2Mana1-3)Manb1-4GlcNAcb1-4(Fuca1-6)GlcNAcb-Sp22	1.2			50.2		81.6	0.3					63.0
415	Galb1-3GlcNAcb1-6(Galb1-3GlcNAcb1-2)Mana1-6(Galb1-3GlcNAcb1-2Mana1-3)Manb1-4GlcNAcb1-4GlcNAcb-Sp19		10.2				88.9	0.5					
416	Galb1-4GlcNAcb1-6(Fuca1-2Galb1-3GlcNAcb1-3)Galb1-4Glc-Sp21		34.8		13.1		92.6	0.5					
417	Fuca1-3GlcNAcb1-6(Galb1-4GlcNAcb1-3)Galb1-4Glc-Sp21							2.9					
418	GlcNAcb1-2Mana1-6(GlcNAcb1-4)(GlcNAcb1-2Mana1-3)Manb1-4GlcNAcb1-4GlcNAc-Sp21												
419	GlcNAcb1-2Mana1-6(GlcNAcb1-4)(GlcNAcb1-4(GlcNAcb1-2)Mana1-3)Manb1-4GlcNAcb1-4GlcNAc-Sp21												
420	GlcNAcb1-6(GlcNAcb1-2)Mana1-6(GlcNAcb1-4)(GlcNAcb1-2Mana1-3)Manb1-4GlcNAcb1-4GlcNAc-Sp21												
421	GlcNAcb1-6(GlcNAcb1-2)Mana1-6(GlcNAcb1-4)(GlcNAcb1-4(GlcNAcb1-2)Mana1-3)Manb1-4GlcNAcb1-4GlcNAc-Sp21												
422	Galb1-4GlcNAcb1-2Mana1-6(GlcNAcb1-4)(Galb1-4GlcNAcb1-2Mana1-3)Manb1-4GlcNAcb1-4GlcNAc-Sp21								49.0				
423	Galb1-4GlcNAcb1-2Mana1-6(GlcNAcb1-4)(Galb1-4GlcNAcb1-4(Galb1-4GlcNAcb1-2)Mana1-3)Manb1-4GlcNAcb1-4GlcNAc-Sp21		7.6					1.0					
424	Galb1-4GlcNAcb1-6(Galb1-4GlcNAcb1-2)Mana1-6(GlcNAcb1-4)(Galb1-4GlcNAcb1-2Mana1-3)Manb1-4GlcNAcb1-4GlcNAc-Sp21		8.4					1.5					
425	Galb1-4GlcNAcb1-6(Galb1-4GlcNAcb1-2)Mana1-6(GlcNAcb1-4)(Galb1-4GlcNAcb1-4(Galb1-4GlcNAcb1-2)Mana1-3)Manb1-4GlcNAcb1-4GlcNAc-Sp21	8.0			7.5		5.9	0.5					
426	Galb1-4Galb-Sp10												
427	Galb1-6Galb-Sp10												
428	Neu5Aca2-3Galb1-4GlcNAcb1-3Galb-Sp8		12.1						25.5				
429	GalNAcb1-6GalNAcb-Sp8												
430	(6S)Galb1-3GlcNAcb-Sp0								9.3				
431	(6S)Galb1-3(6S)GlcNAc-Sp0												
432	Fuca1-2Galb1-4 GlcNAcb1-2Mana1-6(Fuca1-2Galb1-4GlcNAcb1-2(Fuca1-2Galb1-4GlcNAcb1-4)Mana1-3)Manb1-4GlcNAcb1-4GlcNAcb-Sp12		23.9		21.2				35.1		10.2		19.4

433	Fuca1-2Galb1-4(Fuca1-3)GlcNAcb1-2Mana1-6(Fuca1-2Galb1-4(Fuca1-3)GlcNAcb1-4(Fuca1-2Galb1-4(Fuca1-3)GlcNAcb1-2)Mana1-3)Manb1-4GlcNAcb1-4GlcNAcb-Sp12												
434	Galb1-4(Fuca1-3)GlcNAcb1-6GalNAc-Sp14												
435	Galb1-4GlcNAcb1-2Mana-Sp0												
436	Fuca1-2Galb1-4GlcNAcb1-6(Fuca1-2Galb1-4GlcNAcb1-3)GalNAc-Sp14		15.1		8.9			2.3					
437	Gala1-3(Fuca1-2)Galb1-4GlcNAcb1-6(Gala1-3(Fuca1-2)Galb1-4GlcNAcb1-3)GalNAc-Sp14	1.3		7.6			8.5	0.3					48.2
438	GalNAca1-3(Fuca1-2)Galb1-4GlcNAcb1-6(GalNAca1-3(Fuca1-2)Galb1-4GlcNAcb1-3)GalNAc-Sp14	2.2			47.9			0.3			9.8		24.6
439	Neu5Aca2-8Neu5Aca2-3Galb1-3GalNAcb1-4(Neu5Aca2-8Neu5Aca2-3)Galb1-4Glcb-Sp0		22.7		9.3			3.0					
440	GalNAcb1-4Galb1-4Glcb-Sp0												
441	GalNAca1-3(Fuca1-2)Galb1-4GlcNAcb1-2Mana1-6(GalNAca1-3(Fuca1-2)Galb1-4GlcNAcb1-2Mana1-3)Manb1-4GlcNAcb1-4(Fuca1-6)GlcNAcb-Sp22		16.0		9.0			0.3			32.8		17.6
442	Gala1-3(Fuca1-2)Galb1-3GlcNAcb1-2Mana1-6(Gala1-3(Fuca1-2)Galb1-3GlcNAcb1-2Mana1-3)Manb1-4GlcNAcb1-4(Fuca1-6)GlcNAcb-Sp22		21.8		23.6		58.9	0.3			79.9		16.7
443	Neu5Aca2-6Galb1-4GlcNAcb1-6(Fuca1-2Galb1-3GlcNAcb1-3)Galb1-4Glc-Sp21							1.7					
444	GalNAca1-3(Fuca1-2)Galb1-3GlcNAcb1-2Mana1-6(GalNAca1-3(Fuca1-2)Galb1-3GlcNAcb1-2Mana1-3)Manb1-4GlcNAcb1-4(Fuca1-6)GlcNAcb-Sp22		13.5		11.7			0.3		0.7			
445	Galb1-4GlcNAcb1-6(Galb1-4GlcNAcb1-2)Mana1-6(Galb1-4GlcNAcb1-2Mana1-3)Manb1-4GlcNAcb1-4GlcNAcb-Sp19		9.4		11.1			0.5					
446	Neu5Aca2-3Galb1-4GlcNAcb1-4Mana1-6(GlcNAcb1-4)(Neu5Aca2-3Galb1-4GlcNAcb1-4(Neu5Aca2-3Galb1-4GlcNAcb1-2)Mana1-3)Manb1-4GlcNAcb1-4GlcNAcb-Sp21		32.7		25.8		18.2	1.9			6.4		
447	Neu5Aca2-3Galb1-4GlcNAcb1-6(Neu5Aca2-3Galb1-4GlcNAcb1-2)Mana1-6(GlcNAcb1-4)(Neu5Aca2-3Galb1-4GlcNAcb1-2Mana1-3)Manb1-4GlcNAcb1-4GlcNAcb-Sp21		40.8		12.2			2.7					
448	Neu5Aca2-3Galb1-4GlcNAcb1-6(Neu5Aca2-3Galb1-4GlcNAcb1-2)Mana1-6(GlcNAcb1-4)(Neu5Aca2-3Galb1-4GlcNAcb1-4(Neu5Aca2-3Galb1-4GlcNAcb1-2)Mana1-3)Manb1-4GlcNAcb1-4GlcNAcb-Sp21	7.8			19.6		28.5	2.2					
449	Neu5Aca2-6Galb1-4GlcNAcb1-4Mana1-6(GlcNAcb1-4)(Neu5Aca2-6Galb1-4GlcNAcb1-4(Neu5Aca2-6Galb1-4GlcNAcb1-2)Mana1-3)Manb1-4GlcNAcb1-4GlcNAcb-Sp21												
450	Neu5Aca2-6Galb1-4GlcNAcb1-6(Neu5Aca2-6Galb1-4GlcNAcb1-2)Mana1-6(GlcNAcb1-4)(Neu5Aca2-6Galb1-4GlcNAcb1-2Mana1-3)Manb1-4GlcNAcb1-4GlcNAcb-Sp21												
451	Gala1-3(Fuca1-2)Galb1-3GalNAca-Sp8								82.0				
452	Gala1-3(Fuca1-2)Galb1-3GalNAcb-Sp8								79.7				
453	Glca1-6Glca1-6Glca1-6Glcb-Sp10												
454	Glca1-4Glca1-4Glca1-4Glcb-Sp10												
455	Neu5Aca2-3Galb1-4GlcNAcb1-6(Neu5Aca2-3Galb1-4GlcNAcb1-3)GalNAca-Sp14		8.8										
456	Fuca1-2Galb1-4(Fuca1-3)GlcNAcb1-2Mana1-6(Fuca1-2Galb1-4(Fuca1-3)GlcNAcb1-2Mana1-3)Manb1-4GlcNAcb1-4(Fuca1-6)GlcNAcb-Sp24								9.6				
457	Fuca1-2Galb1-3(Fuca1-4)GlcNAcb1-2Mana1-6(Fuca1-2Galb1-3(Fuca1-4)GlcNAcb1-2Mana1-3)Manb1-4GlcNAcb1-4(Fuca1-6)GlcNAcb1-4(Fuca1-6)GlcNAcb-Sp19												
458	GlcNAcb1-6(GlcNAcb1-2)Mana1-6(GlcNAcb1-2Mana1-3)Manb1-4GlcNAcb1-4(Fuca1-6)GlcNAcb-Sp24								17.8				
459	Galb1-3GlcNAcb1-2Mana1-6(GlcNAcb1-4)(Galb1-3GlcNAcb1-2Mana1-3)Manb1-4GlcNAcb1-4GlcNAcb-Sp21						6.9	1.4					
460	Neu5Aca2-6Galb1-4GlcNAcb1-6(Galb1-3GlcNAcb1-3)Galb1-4Glcb-Sp21							2.7					
461	Neu5Aca2-3Galb1-4GlcNAcb1-2Mana-Sp0												
462	Neu5Aca2-3Galb1-4GlcNAcb1-6GalNAca-Sp14		7.9										
463	Neu5Aca2-6Galb1-4GlcNAcb1-6GalNAca-Sp14												
464	Neu5Aca2-6Galb1-4 GlcNAcb1-6(Neu5Aca2-6Galb1-4GlcNAcb1-3)GalNAca-Sp14												
465	Neu5Aca2-6Galb1-4GlcNAcb1-2Mana1-6(Neu5Aca2-6Galb1-4GlcNAcb1-2Mana1-3)Manb1-4GlcNAcb1-4(Fuca1-6)GlcNAcb-Sp24												

466	Neu5Aca2-3Galb1-4GlcNAcb1-2Mana1-6(Neu5Aca2-3Galb1-4GlcNAcb1-2Mana1-3)Manb1-4GlcNAcb1-4(Fuca1-6)GlcNAcb-Sp24		30.7		14.4			2.5					
467	Mana1-6(Mana1-3)Manb1-4GlcNAcb1-4(Fuca1-6)GlcNAcb-Sp19												
468	Galb1-4GlcNAcb1-6(Galb1-4GlcNAcb1-2)Mana1-6(Galb1-4GlcNAcb1-2Mana1-3)Manb1-4GlcNAcb1-4(Fuca1-6)GlcNAcb-Sp24		28.8		12.7			0.4					8.7
469	Neu5Aca2-3Galb1-3GlcNAcb1-2Mana1-6(GlcNAcb1-4)(Neu5Aca2-3Galb1-3GlcNAcb1-2Mana1-3)Manb1-4GlcNAcb1-4GlcNAc-Sp21												
470	Neu5Aca2-6Galb1-4GlcNAcb1-6(Fuca1-2Galb1-4(Fuca1-3)GlcNAcb1-3)Galb1-4Glc-Sp21												
471	Galb1-3GlcNAcb1-6GalNAca-Sp14							1.8					
472	Gala1-3Galb1-3GlcNAcb1-6GalNAca-Sp14		20.2		10.0		24.2	0.5					
473	Galb1-3(Fuca1-4)GlcNAcb1-6GalNAca-Sp14								7.9				
474	Neu5Aca2-3Galb1-3GlcNAcb1-6GalNAca-Sp14								29.2				
475	(3S)Galb1-3(Fuca1-4)GlcNAcb-Sp0												
476	Galb1-4(Fuca1-3)GlcNAcb1-6(Neu5Aca2-6(Neu5Aca2-3Galb1-3)GlcNAcb1-3)Galb1-4Glc-Sp21								5.6				
477	Fuca1-2Galb1-4GlcNAcb1-6GalNAca-Sp14		9.0						26.9				
478	Gala1-3Galb1-4GlcNAcb1-6GalNAca-Sp14		30.5		14.6			1.3					
479	Galb1-4(Fuca1-3)GlcNAcb1-2Mana-Sp0												
480	Fuca1-2(6S)Galb1-3GlcNAcb-Sp0												
481	Gala1-3(Fuca1-2)Galb1-4GlcNAcb1-6GalNAca-Sp14												
482	Fuca1-2Galb1-4GlcNAcb1-2Mana-Sp0												
483	Fuca1-2Galb1-3(6S)GlcNAcb-Sp0		10.9				9.5	2.2					
484	Fuca1-2(6S)Galb1-3(6S)GlcNAcb-Sp0								10.3				
485	Neu5Aca2-6GalNAcb1-4(6S)GlcNAcb-Sp8								6.1				
486	GalNAcb1-4(Fuca1-3)(6S)GlcNAcb-Sp8												
487	(3S)GalNAcb1-4(Fuca1-3)GlcNAcb-Sp8												
488	Fuca1-2Galb1-3GlcNAcb1-6(Fuca1-2Galb1-3GlcNAcb1-3)GalNAca-Sp14						70.1	1.8					
489	GalNAca1-3(Fuca1-2)Galb1-3GlcNAcb1-6GalNAca-Sp14		44.4		27.1			0.3		1.6			
490	GlcNAcb1-6(GlcNAcb1-2)Mana1-6(GlcNAcb1-4)(GlcNAcb1-4(GlcNAcb1-2)Mana1-3)Manb1-4GlcNAcb1-4(Fuca1-6)GlcNAc-Sp21												
491	Galb1-4GlcNAcb1-6(Galb1-4GlcNAcb1-2)Mana1-6(GlcNAcb1-4)Galb1-4GlcNAcb1-4(Gal b1-4GlcNAcb1-2)Mana1-3)Manb1-4GlcNAcb1-4(Fuca1-6)GlcNAc-Sp21		52.7		50.9			0.3					14.0
492	Galb1-3GlcNAca1-3Galb1-4GlcNAcb-Sp8		9.8				13.6	0.3					
493	Galb1-3(6S)GlcNAcb-Sp8												
494	(6S)(4S)GalNAcb1-4GlcNAc-Sp8												
495	(6S)GalNAcb1-4GlcNAc-Sp8												
496	(3S)GalNAcb1-4(3S)GlcNAc-Sp8								6.0				
497	GalNAcb1-4(6S)GlcNAc-Sp8							1.8					
498	(3S)GalNAcb1-4GlcNAc-Sp8		21.6		16.6		12.2	1.1					
499	(4S)GalNAcb-Sp10												
500	Galb1-4(6P)GlcNAcb-Sp0												
501	(6P)Galb1-4GlcNAcb-SP0												
502	GalNAca1-3(Fuca1-2)Galb1-4GlcNAcb1-6GalNAc-Sp14	3.2			35.6			0.4					
503	Neu5Aca2-6Galb1-4GlcNAcb1-2Man-Sp0												
504	Gala1-3Galb1-4GlcNAcb1-2Mana-Sp0	2.4			29.7			0.5					
505	GalNAca1-3(Fuca1-2)Galb1-4 GlcNAcb1-2Mana-Sp0	1.1			66.7			0.2		2.6			42.7
506	Galb1-3GlcNAcb1-2Mana-Sp0						13.9	3.0					
507	Gala1-3(Fuca1-2)Galb1-3GlcNAcb1-6GalNAc-Sp14	1.8			36.5		100.0	0.3					22.3
508	Neu5Aca2-3Galb1-3GlcNAcb1-2Mana-Sp0		9.6				39.8		80.1				
509	Gala1-3Galb1-3GlcNAcb1-2Mana-Sp0	9.7			17.9		36.1	0.5					
510	GalNAcb1-4GlcNAcb1-2Mana-Sp0							2.0					
511	Neu5Aca2-3Galb1-3GlcNAcb1-4Galb1-4Glcb-Sp0												
512	GlcNAcb1-2 Mana1-6(GlcNAcb1-4)(GlcNAcb1-2Mana1-3)Manb1-4GlcNAcb1-4(Fuca1-6)GlcNAc-Sp21												
513	Galb1-4GlcNAcb1-2 Mana1-6(GlcNAcb1-4)(Galb1-4GlcNAcb1-2Mana1-3)Manb1-4GlcNAcb1-4(Fuca1-6)GlcNAc-Sp21		23.2		9.3			0.4					
514	Galb1-4GlcNAcb1-2 Mana1-6(Galb1-4GlcNAcb1-4)(Galb1-4GlcNAcb1-2Mana1-3)Manb1-4GlcNAcb1-4(Fuca1-6)GlcNAc-Sp21							0.5					
515	Fuca1-4(Galb1-3)GlcNAcb1-2 Mana-Sp0												
516	Neu5Aca2-3Galb1-4(Fuca1-3)GlcNAcb1-2Mana-Sp0												
517	GlcNAcb1-3Galb1-4GlcNAcb1-6(GlcNAcb1-3)Galb1-4GlcNAc-Sp0	9.8			9.9			0.3					

518	GalNAca1-3(Fuca1-2)Galb1-3GalNAcb1-3Gala1-4Galb1-4Glc-Sp21							2.0					
519	Gala1-3(Fuca1-2)Galb1-3GalNAcb1-3Gala1-4Galb1-4Glc-Sp21								49.7				
520	Galb1-3GalNAcb1-3Gal-Sp21							1.4					
521	GlcNAcb1-3Galb1-4GlcNAcb1-2Mana1-6(GlcNAcb1-3Galb1-4GlcNAcb1-2Mana1-3)Manb1-4GlcNAcb1-4GlcNAcb-Sp12							0.3			11.0		24.4
522	Galb1-4GlcNAcb1-3Galb1-4GlcNAcb1-2Mana1-6(Galb1-4GlcNAcb1-3Galb1-4GlcNAcb1-2Mana1-3)Manb1-4GlcNAcb1-4GlcNAcb-Sp12							2.3					8.8
523	GlcNAcb1-3Galb1-4GlcNAcb1-3Galb1-4GlcNAcb1-2Mana1-6(GlcNAcb1-3Galb1-4GlcNAcb1-3Galb1-4GlcNAcb1-2Mana1-3)Manb1-4GlcNAcb1-4GlcNAcb-Sp12	5.0			23.2			0.4					25.6
524	Galb1-4GlcNAcb1-3Galb1-4GlcNAcb1-3Galb1-4GlcNAcb1-2Mana1-6(Galb1-4GlcNAcb1-3Galb1-4GlcNAcb1-3Galb1-4GlcNAcb1-2Mana1-3)Manb1-4GlcNAcb1-4GlcNAcb-Sp12	2.4			56.1		34.8	0.3			66.6		63.7
525	Galb1-3GlcNAcb1-3Galb1-4GlcNAcb1-2Mana1-6(Galb1-3GlcNAcb1-3Galb1-4GlcNAcb1-2Mana1-3)Manb1-4GlcNAcb1-4GlcNAc-Sp25	5.5			33.1		27.0	0.3			21.3		38.8
526	Neu5Gca2-8Neu5Gca2-3Galb1-4GlcNAc-Sp0		28.9		9.2				10.8				
527	Neu5Aca2-8Neu5Gca2-3Galb1-4GlcNAc-Sp0	1.7			7.6			2.4					
528	Neu5Gca2-8Neu5Aca2-3Galb1-4GlcNAc-Sp0		18.5					2.2					
529	Neu5Gca2-8Neu5Gca2-3Galb1-4GlcNAcb1-3Galb1-4GlcNAc-Sp0		27.7		25.0		7.3	2.4					
530	Neu5Gca2-8Neu5Gca2-6Galb1-4GlcNAc-Sp0												
531	Neu5Aca2-8Neu5Aca2-3Galb1-4GlcNAc-Sp0	3.3											
532	GlcNAcb1-3Galb1-4GlcNAcb1-6(GlcNAcb1-3Galb1-4GlcNAcb1-2)Mana1-6(GlcNAcb1-3Galb1-4GlcNAcb1-2Man a1-3)Manb1-4GlcNAcb1-4GlcNAc-Sp24	6.3			40.3			0.3			15.4		67.7
533	Galb1-4GlcNAcb1-3Galb1-4GlcNAcb1-6(Galb1-4GlcNAcb1-3Galb1-4GlcNAcb1-2)Mana1-6(Galb1-4GlcNAcb1-3Galb1-4GlcNAcb1-2Mana1-3)Manb1-4GlcNAcb1-4GlcNAc-Sp24	6.8			23.8		17.4	0.4			18.2		23.8
534	Gala1-3Galb1-4GlcNAcb1-2Mana1-6(Gala1-3Galb1-4GlcNAcb1-2Mana1-3)Manb1-4GlcNAcb1-4GlcNAc-Sp24	2.5			75.9		76.5	0.3					24.9
535	GlcNAcb1-3Galb1-4GlcNAcb1-6(GlcNAcb1-3Galb1-3)GalNAca-Sp14		11.4					0.7					
536	GalNAcb1-3GlcNAcb-Sp0		8.1				64.5	1.0					
537	GalNAcb1-4GlcNAcb1-3GalNAcb1-4GlcNAcb-Sp0	2.2			69.5		13.4	0.9					
538	GlcNAb1-3Galb1-3GalNAc-Sp14								86.1				
539	Galb1-3GlcNAcb1-6(Galb1-3)GalNAc-Sp14		6.5				78.9	0.2					58.0
540	(3S)GlcAb1-3Galb1-4GlcNAcb1-3Galb1-4Glc-Sp0	1.7			50.8		90.4	1.5			6.6		
541	(3S)GlcAb1-3Galb1-4GlcNAcb1-2Mana-Sp0	2.6			40.4		53.4		72.8				
542	Galb1-3GlcNAcb1-3Galb1-4GlcNAcb1-3Galb1-4GlcNAcb1-6(Galb1-3GlcNAcb1-3Galb1-4GlcNAcb1-3Galb1-4GlcNAb1-2)Mana1-6(Galb1-3GlcNAcb1-3Galb1-4GlcNAcb1-3Galb1-4GlcNAcb1-2Mana1-3)Manb1-4GlcNAcb1-4(Fuca1-6)GlcNAcb-Sp24	3.1			46.4		50.2	0.3			23.2	3.0	
543	Galb1-3GlcNAcb1-3Galb1-4GlcNAcb1-6(Galb1-3GlcNAcb1-3Galb1-4GlcNAb1-2)Mana1-6(Galb1-3GlcNAcb1-3Galb1-4GlcNAcb1-2Mana1-3)Manb1-4GlcNAcb1-4(Fuca1-6)GlcNAcb-Sp24						60.3	0.3			16.6		54.8
544	Galb1-4GlcNAcb1-3Galb1-4GlcNAcb1-3GalNAca-Sp14	8.2			17.9			0.4					
545	Galb1-4GlcNAcb1-3Galb1-4GlcNAcb1-6(Galb1-3)GalNAca-Sp14		32.4		19.0			0.4					
546	Galb1-4GlcNAcb1-3Galb1-4GlcNAcb1-6(Galb1-4GlcNAcb1-3Galb1-4GlcNAcb1-3)GalNAca-Sp14	9.0			34.1		34.6	0.4			6.3		10.0
547	GlcNAcb1-3Galb1-4GlcNAcb1-3GalNAca-Sp14		19.9		15.5			0.7					
548	GlcNAcb1-3Galb1-4GlcNAcb1-6(Galb1-3)GalNAca-Sp14		17.0		11.4			1.1					
549	GlcNAcb1-3Galb1-4GlcNAcb1-6(GlcNAcb1-3Galb1-4GlcNAcb1-3)GalNAca-Sp14	8.5			28.0			0.5					
550	GlcNAcb1-3Galb1-4GlcNAcb1-3Galb1-4GlcNAcb1-3GalNAca-Sp14	4.7			38.9			0.3					16.2
551	Galb1-4GlcNAcb1-3Galb1-3GalNAca-Sp14							0.4			15.9		
552	Neu5Aca2-6Galb1-4GlcNAcb1-6(Galb1-3)GalNAca-Sp14								44.6				
553	GlcNAcb1-6(Neu5Aca2-3Galb1-3)GalNAca-Sp14								30.0				
554	Galb1-3GalNAcb1-4(Neu5Aca2-8Neu5Aca2-8Neu5Aca2-3)Galb1-4Glcb-Sp21												

555	Fuca1-2Galb1-4GlcNAcb1-3Galb1-4GlcNAcb1-2Mana1-6(Fuca1-2Galb1-4GlcNAcb1-3Galb1-4GlcNAcb1-2Mana1-3)Manb1-4GlcNAcb1-4GlcNAcb-Sp24	NA	NA	NA	NA	0.1		0.3					
