# Supplementary material for: An Automated Approach to Assess Relative Galectin-Glycan Affinity Following Glycan Microarray Analysis
Source: Front Mol Biosci. 2022 Aug 11;9:893185. doi: 10.3389/fmolb.2022.893185 (PMC9403319; doi:10.3389/fmolb.2022.893185)
Supplement: Supplementary file 2 [file DataSheet1.pdf]

Supplementary Material

Supplementary Figure 1

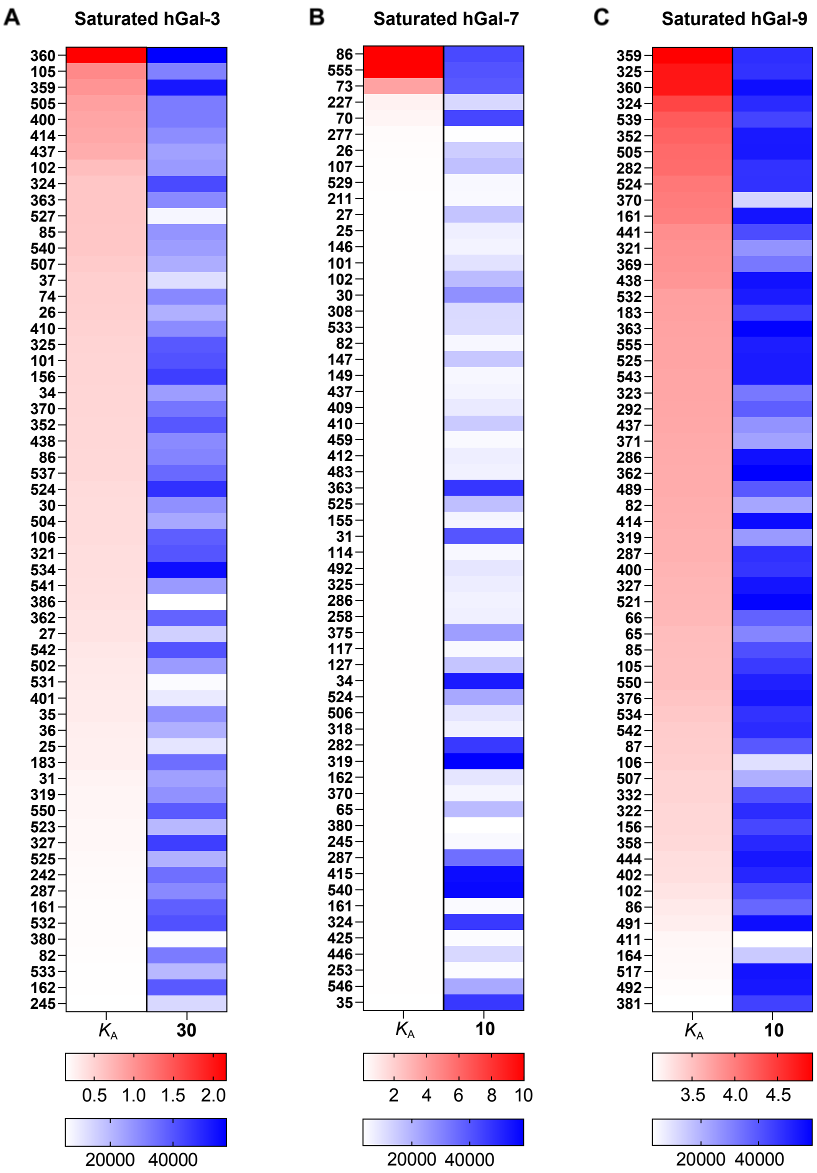

Supplement Figure 1. Alternate examination of K_A and RFU correlation for selected galectin. (A) A correlation map with the top 60 samples' K_A and RFU values, sorted by K_A, for Gal-3. (B) A correlation map with the top 60 samples' K_A and RFU values, sorted by K_A, for Gal-7. (C) A correlation map with the top 60 samples' K_A and RFU values, sorted by K_A, for Gal-9. There are two sets of gradients: red and blue to indicate binding strength, for K_A and RFU, respectively. For both gradients, darker tint = higher calculated value, lighter tint = lower calculated value.

Supplementary Figure 2

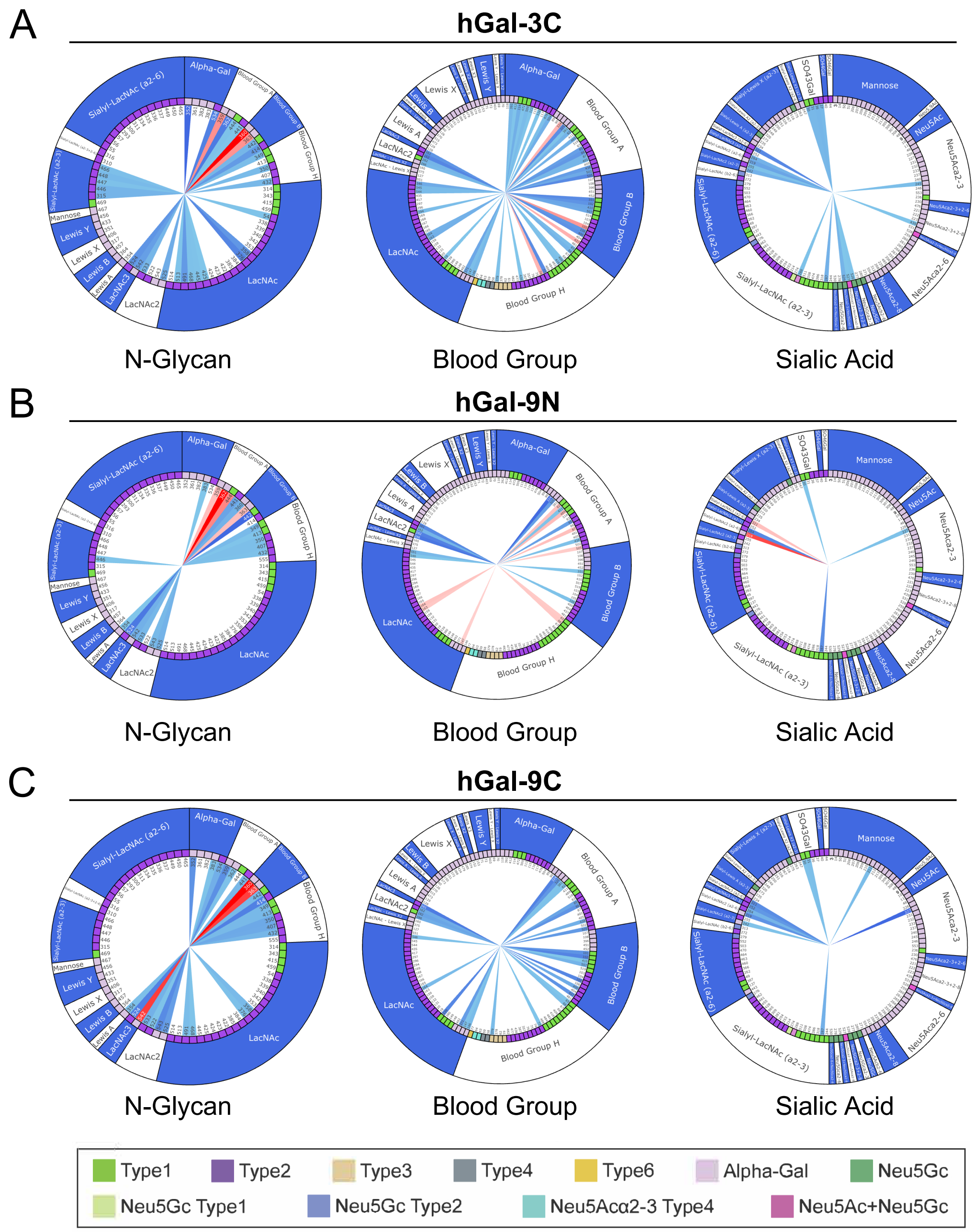

Supplement Figure 2. Additional demonstration of kaPlotting's nested pie charts. Nested pie charts shown for Gal-3C (A), Gal-9N (B) and Gal-9C (C). Charts are grouped by N-Glycan, Lewis Antigen, and Sialic Acid, and plotted using kaPlotting. Each nested pie chart has three layers of information: K_A, glycan linkage, and glycan structure, from inner to outer, respectively. There are two sets of gradients: red and blue, that represent K_A and % max on the inner circle. For both gradients, darker tint = higher the calculated value, lighter tint = lower calculated value. Legend at the bottom shows the glycan linkage (second layer on the nested pie charts).

Supplementary Table 1. Galectin binding to glycans present on the CFG microarray. The data table has the complete list of 555 glycan structures and their corresponding calculated K_A and % max for Gal-3, Gal-3C, Gal-7, Gal-9, Gal-9N, and Gal-9C. The percent binding compared to the maximum binding is shown as % max.
